# Refining epigenetic prediction of chronological and biological age

**DOI:** 10.1186/s13073-023-01161-y

**Published:** 2023-02-28

**Authors:** Elena Bernabeu, Daniel L. McCartney, Danni A. Gadd, Robert F. Hillary, Ake T. Lu, Lee Murphy, Nicola Wrobel, Archie Campbell, Sarah E. Harris, David Liewald, Caroline Hayward, Cathie Sudlow, Simon R. Cox, Kathryn L. Evans, Steve Horvath, Andrew M. McIntosh, Matthew R. Robinson, Catalina A. Vallejos, Riccardo E. Marioni

**Affiliations:** 1 Centre for Genomic and Experimental Medicine, Institute of Genetics and Cancer, University of Edinburgh, Edinburgh, UK; 2 Department of Human Genetics, David Geffen School of Medicine, University of California, Los Angeles, CA USA; 3 Altos Labs, San Diego, USA; 4 Edinburgh Clinical Research Facility, University of Edinburgh, Edinburgh, UK; 5 Department of Psychology, Lothian Birth Cohorts, University of Edinburgh, Edinburgh, UK; 6 Medical Research Council Human Genetics Unit, Institute of Genetics and Cancer, University of Edinburgh, Edinburgh, UK; 7 Centre for Clinical Brain Sciences, University of Edinburgh, Edinburgh, UK; 8 BHF Data Science Centre, Health Data Research UK, London, UK; 9 Edinburgh Medical School, Usher Institute, University of Edinburgh, Edinburgh, UK; 10 Division of Psychiatry, University of Edinburgh, Royal Edinburgh Hospital, Edinburgh, UK; 11 Institute of Science and Technology Austria, Klosterneuburg, Austria; 12 The Alan Turing Institute, London, UK

## Abstract

**Background:**

Epigenetic clocks can track both chronological age (cAge) and biological age (bAge). The latter is typically defined by physiological biomarkers and risk of adverse health outcomes, including all-cause mortality. As cohort sample sizes increase, estimates of cAge and bAge become more precise. Here, we aim to develop accurate epigenetic predictors of cAge and bAge, whilst improving our understanding of their epigenomic architecture.

**Methods:**

First, we perform large-scale (*N* = 18,413) epigenome-wide association studies (EWAS) of chronological age and all-cause mortality. Next, to create a cAge predictor, we use methylation data from 24,674 participants from the Generation Scotland study, the Lothian Birth Cohorts (LBC) of 1921 and 1936, and 8 other cohorts with publicly available data. In addition, we train a predictor of time to all-cause mortality as a proxy for bAge using the Generation Scotland cohort (1214 observed deaths). For this purpose, we use epigenetic surrogates (EpiScores) for 109 plasma proteins and the 8 component parts of GrimAge, one of the current best epigenetic predictors of survival. We test this bAge predictor in four external cohorts (LBC1921, LBC1936, the Framingham Heart Study and the Women’s Health Initiative study).

**Results:**

Through the inclusion of linear and non-linear age-CpG associations from the EWAS, feature pre-selection in advance of elastic net regression, and a leave-one-cohort-out (LOCO) cross-validation framework, we obtain cAge prediction with a median absolute error equal to 2.3 years. Our bAge predictor was found to slightly outperform GrimAge in terms of the strength of its association to survival (HR_GrimAge_ = 1.47 [1.40, 1.54] with *p* = 1.08 × 10^−52^, and HR_bAge_ = 1.52 [1.44, 1.59] with *p* = 2.20 × 10^−60^). Finally, we introduce MethylBrowsR, an online tool to visualise epigenome-wide CpG-age associations.

**Conclusions:**

The integration of multiple large datasets, EpiScores, non-linear DNAm effects, and new approaches to feature selection has facilitated improvements to the blood-based epigenetic prediction of biological and chronological age.

**Supplementary Information:**

The online version contains supplementary material available at 10.1186/s13073-023-01161-y.

## Background

The development and application of epigenetic predictors for healthcare research has grown dramatically over the last decade [1]. These predictors can aid disease risk stratification and are based on associations between CpG DNA methylation (DNAm) and age, health, and lifestyle outcomes. DNAm is dynamic, tissue-specific, and is influenced by both genetic and environmental factors. DNAm can precisely track ageing through predictors termed “epigenetic clocks” [2–8]. DNAm has also been found to capture other components of health, such as smoking status [9, 10], alcohol consumption [11, 12], obesity [11, 13], and protein levels [14].

“First generation” epigenetic ageing clocks, including those by Horvath [3] and Hannum et al. [4], were trained on chronological age [2–4] (cAge), with near-perfect clocks expected to arise as sample sizes grow [5]. However, cAge clocks hold limited capability for tracking and quantifying age-related health status, also termed biological age (bAge) [5, 8]. To address this, “second generation” clocks have been trained on other age-related measures, including a phenotypic biomarker of morbidity (PhenoAge [15]), rate of ageing (DunedinPACE [16]), and time to all-cause mortality (GrimAge [17]). Regressing an epigenetic clock predictor (whether trained on cAge or bAge) on chronological age within a cohort gives rise to an “age acceleration” residual with positive values corresponding to faster biological ageing.

Penalised regression approaches such as elastic net [18] are commonly used to derive epigenetic predictors. These identify a weighted linear combination of CpGs that optimally predict an outcome from a statistical perspective, i.e. no preference is given to the location or possible biological role of the input features. The majority consider genome-wide CpG sites as potential predictive features. However, others have used a two-stage approach that first creates DNAm surrogates (or epigenetic scores—EpiScores) for biomarkers (also typically via elastic net) prior to training a second elastic net model on the phenotypic outcome or time to event (TTE) [14, 17]. GrimAge is currently considered one of the best bAge epigenetic clocks [16]. It is derived from age, sex, and EpiScores of smoking pack years and seven plasma proteins that have been associated with mortality or morbidity: adrenomedullin (ADM), beta-2-microglobulin (B2M), cystatin C, growth differentiation factor 15 (GDF15), leptin, plasminogen activation inhibitor 1 (PAI1), and tissue inhibitor metalloproteinase (TIMP1). Recently, a wider set of 109 EpiScores for the circulating proteome were generated by Gadd et al. [14]. These have not yet been considered as potential features for the prediction of bAge.

**Fig. 1:**
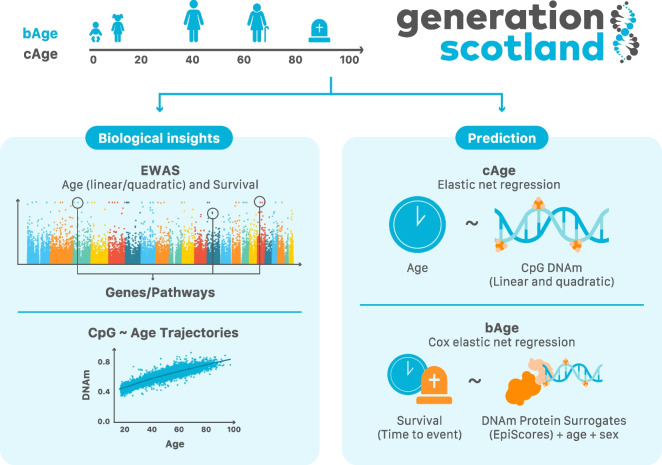
Study overview. Using the Generation Scotland cohort as our main data source, we explored the relationship between the epigenome and age/survival via EWAS, which also informed on genes of interest and potentially enriched pathways. We further characterised epigenome-wide CpG ~ age trajectories, which can be visualised in a new Shiny app, MethylBrowsR (https://shiny.igmm.ed.ac.uk/MethylBrowsR/). Finally, we refined epigenetic prediction of both cAge and bAge. Calculation of cAge can be performed either using a standalone script (https://github.com/elenabernabeu/cage_bage/tree/main/cage_predictor) or by uploading DNAm data to our MethylDetectR shiny app (https://shiny.igmm.ed.ac.uk/MethylDetectR/). As the weights for GrimAge and its component parts are not publicly available, bAge can only be calculated by using our standalone script (https://github.com/elenabernabeu/cage_bage/tree/main/bage_predictor), after obtaining GrimAge estimates from an external online calculator (http://dnamage.genetics.ucla.edu/new)

Here, we sought to improve the prediction of both cAge and bAge (Fig. 1). We first present large-scale epigenome-wide association studies (EWAS) of cAge (for both linear and quadratic CpG effects) and time to all-cause mortality as a proxy for bAge. A predictor of cAge is then generated using DNAm data from 11 cohorts, including samples from > 18,000 participants of the Generation Scotland study [19]. We use a leave-one-cohort-out (LOCO) prediction framework, including feature pre-selection ahead of elastic net for linear and non-linear DNAm-age relationships (ascertained through the EWAS), to test its performance. Through data linkage to death records in Generation Scotland, we develop a bAge predictor of time to all-cause mortality, which we compare against GrimAge, in four external cohorts. These analyses highlight the potential for large DNAm resources to generate increasingly accurate predictors of (i) cAge, with potential forensic utility, and (ii) bAge, with potential implications for risk prediction and clinical trials.

## Methods

### Data overview

Generation Scotland is a Scottish family-based study with over 24,000 participants recruited between 2006 and 2011 [19]. Blood-based DNAm levels at 752,722 CpG sites were quantified using the Illumina MethylationEPIC array for 18,413 individuals. Participants were aged between 18 and 99 years at recruitment, with a mean age of 47.5 years (SD 14.9, Table 1). The data was processed in three sets (*N*_Set1_ = 5087, *N*_Set2_ = 4450, *N*_Set3_ = 8876), with a total of 121 experimental batches (see Additional file 1).

**Table 1:** Age profile and test set prediction performance for cohorts used in cAge predictor training and testing. Predictions were made using a LOCO approach, where each cohort was excluded in training and the resulting model was used for testing (see Methods). Models were trained on age, and if an individual was predicted to be under 20, their prediction was re-estimated considering models trained on log(age). External cohort information taken from Zhang et al. [5]. *r* column states Pearson correlation, RMSE the root mean squared error, and MAE the median absolute error

						**Prediction accuracy**		
**Cohort**	***N***	**Mean age (SD)**	**Age range**	***N***_**Females**_** (%)**	**Tissue**	***r***	**RMSE**	**MAE**
GS	18,413	47.5 (14.9)	[17.1, 98.5]	10,833 (58.8%)	Blood	-	-	-
LBC1921^20,21^	692	82.3 (4.3)	[77.8,90.6]	401 (57.9%)	Blood	0.659	4.050	2.466
LBC1936^20,21^	2796	73.6 (3.7)	[67.7,80.9]	1356 (48.5%)	Blood	0.685	3.311	2.099
GSE72775^22^	335	70.2 (10.3)	[36.5, 90.5]	138 (41.2%)	Blood	0.949	3.275	1.843
GSE78874^22^	259	68.8 (9.7)	[36.0, 88.0]	113 (43.6%)	Saliva	0.875	6.826	4.333
GSE72773^22^	310	65.6 (13.9)	[35.1, 91.9]	150 (48.4%)	Blood	0.945	4.611	2.068
GSE72777^22^	46	14.7 (10.4)	[2.2, 35.0]	31 (67.4%)	Blood	0.942	4.211	2.505
GSE41169^a,23^	95	31.6 (10.3)	[18.0, 65.0]	28 (29.5%)	Blood	0.975	2.869	1.947
GSE40279^4^	656	64.0 (14.7)	[19.0, 101.0]	338 (51.5%)	Blood	0.969	3.697	2.074
GSE42861^a,24^	689	51.9 (11.8)	[18.0, 70.0]	492 (71.4%)	Blood	0.972	4.498	3.563
GSE53740^a,25^	383	67.8 (9.6)	[34.0, 93.0]	155 (40.5%)	Blood	0.921	4.443	2.797

^a^Some cohorts contain case/control data. GSE41169: schizophrenia 62, control 33; GSE42861: rheumatoid arthritis 354, control 335; GSE53740: Alzheimer’s disease 15, corticobasal degeneration 1, frontotemporal dementia (FTD) 121, FTD/MND 7, progressive supranuclear palsy 43, control 193, unknown 4

In order to train and test a cAge predictor, Generation Scotland data as well as that from an additional 6261 individuals from ten external cohorts were considered. These included the Lothian Birth Cohorts (LBC) of 1921 and 1936 [20, 21] and eight publicly available Gene Expression Omnibus (GEO) datasets (Table 1) [4, 22–25]. In addition, the independent dataset GEO GSE55763 [13, 26] (2711 samples from 2664 individuals) was used to assess cAge clock performance against existing clocks in individuals not used for training across any of the predictors considered. Given that the external datasets assessed DNAm (blood-based apart from GSE78874, which considered saliva) using the Illumina HumanMethylation450K array, the Generation Scotland data were subset to 374,791 CpGs that were present across all studies. Missing values were mean imputed per CpG and per cohort.

The bAge predictor was trained using data for 18,365 participants from the Generation Scotland cohort for which valid death status data (i.e. death status non-missing, and age at death not lower than age at baseline) via linkage to the National Health Service Central Register was available. A total of 1214 participant deaths have been recorded as of March 2022, when records were last updated. Alive individuals in March 2022 were censored at their age at that time (TTE thus being age in March 2022 minus age at baseline). Average TTE amongst deaths was 7.79 (SD 3.54) years, and average TTE amongst censored samples was 12.82 (SD 1.35) years. To test the bAge predictor, data from an additional 4134 individuals (with a total of 1653 deaths) from four external cohorts (six datasets) were considered. These included the baseline samples of both the LBC1921 and LBC1936 cohorts, as well as the Framingham Heart Study (FHS) [27–29] and the Women’s Health Initiative (WHI) [30, 31] Broad Agency Award 23 (B23) study for Black, White, and Hispanic individuals (Table 2).

**Table 2:** Cox proportional hazards output for GrimAgeAccel and bAgeAccel in the test datasets. Hazard ratios are presented per standard deviation of the GrimAgeAccel and bAgeAccel variables. Further details in Additional File 4: Table S11. Asterisk symbol (*) indicates the following: the FHS cohort used here was the same as the test set from the original GrimAge paper

Cohort	*N*	*N* deaths	GrimAgeAccel Hazard ratio (95% CI)	bAgeAccel Hazard ratio (95% CI)
LBC1936^20,21^	895	367	1.74 (1.57, 1.94)	1.73 (1.56, 1.91)
LBC1921^20,21^	421	421	1.33 (1.20, 1.47)	1.44 (1.29, 1.59)
FHS^*27,28^	711	100	1.72 (1.35, 2.19)	1.77 (1.40, 2.25)
WHI B23 White^30,31^	998	418	1.44 (1.31, 1.58)	1.45 (1.32, 1.60)
WHI B23 Black^30,31^	676	229	1.35 (1.19, 1.53)	1.42 (1.24, 1.62)
WHI B23 Hispanic^30,31^	433	118	1.41 (1.18, 1.68)	1.44 (1.21, 1.72)

A detailed description of the datasets used (Generation Scotland, GEO, LBC, FHS, and WHI) can be found in Additional file 1.

### Epigenome-wide association study of chronological age

We conducted an EWAS to identify CpG sites that had linear or quadratic associations with chronological age, using Generation Scotland data (*N* = 18,413, CpGs = 752,722). Linear regression analyses were carried out which included both linear and quadratic CpG M-values as independent variables and age as the dependent variable (Age ~ CpG and Age ~ CpG + CpG^2^, respectively). Fixed effect covariates included estimated white blood cell (WBC) proportions (basophils, eosinophils, natural killer cells, monocytes, CD4T, and CD8T cells) calculated in the *minfi* R package (version 1.36.0) [32] using the Houseman method [33], sex, DNAm batch/set, smoking status (a factor with 5 levels: current, gave up in the last year, gave up more than a year ago, never, or unknown), smoking pack years (number of packs of cigarettes smoked per day, 20 cigarettes per pack, multiplied by the number of years the person smoked), and 20 DNAm principal components (PCs) to correct for unmeasured confounders. Family structure was not accounted for given the nature of the phenotype. Age was centred by its mean, and CpG/CpG^2^ M-values were scaled to mean zero and variance one. Epigenome-wide significance was set at *p*-value < 3.6 × 10^−8^, as per Saffari et al. [34]. For each CpG-age association, *F*-tests were used to compare models including the CpG as a linear term, versus one including both linear and quadratic terms, whilst controlling for all covariates listed here.

### Epigenome-wide association study of time to all-cause mortality

We conducted an EWAS to identify CpG sites (from a total of 752,722 loci) that were associated with time to all-cause mortality in Generation Scotland. Cox proportional hazards (Cox PH) regression models were fit for each CpG site as the predictor of interest using the *coxph* function from the *survival* R package (version 3.3.1), with time to all-cause mortality or censoring as the survival outcome. Fixed effect covariates included those used in the cAge EWAS (age at baseline, sex, batch/set, smoking status, smoking pack years, WBC estimates, and top 20 DNAm PCs). Epigenome-wide significance was set at *p*-value < 3.6 × 10^−8^.

To assess whether relatedness in the cohort influenced the results, we fit a Cox PH model with a kinship matrix for each significantly associated CpG, using the *coxme* R package (version 2.2.16).

### Prediction of chronological age

We used elastic net regression to derive a predictor of chronological age from the 374,791 CpG sites common across all cohorts considered in cAge training (description of cohorts in Table 1). The L_1_, L_2_ mixing parameter was set at *α* = 0.5 based on epigenetic clock precedent [3, 5]. The *biglasso* R package (version 1.5.1) was used [35], with 25-fold cross-validation (CV; ~ 1000 individuals per fold) to select the shrinkage parameter (*λ*) that minimised the mean cross-validated prediction error. A sensitivity analysis was performed, assigning individuals from the same methylation set, batch, and cohort to individual folds, which returned highly similar results.

The effect of including external cohorts in training, as well as accounting for non-linear relationships and pre-selection of features, amongst others, is briefly detailed in Additional file 2. As a result of these analyses, we created a predictor making use of a LOCO framework, training on both log(age) and age, and performing feature pre-selection ahead of elastic net. Here, we describe each of these steps.

#### Leave-one-cohort-out

**Fig. 2:**
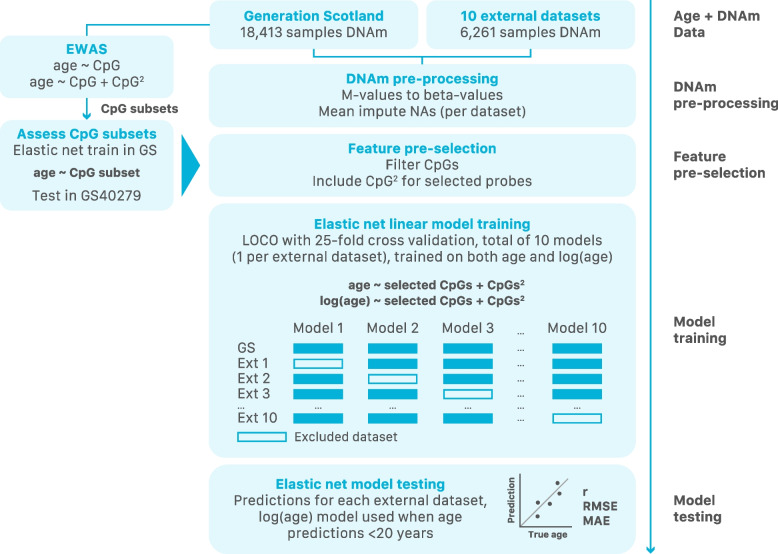
Flowchart for the creation of the cAge predictor. First, DNAm data originating from Generation Scotland and 10 external datasets was pre-processed. Next, CpGs were pre-selected based on the Generation Scotland EWAS for epigenome-wide significant linear and quadratic CpG-age associations. Elastic net models were then trained and tested on the remaining features using a LOCO framework with 25-fold CV, with training on both age and log(age) as outcomes

cAge predictors were created using a LOCO framework where, for each of the 10 external cohorts, a model was trained in Generation Scotland and all but one of the external cohorts (Fig. 2). We then tested each of the 10 trained models on the excluded cohort. A final model was trained using all 11 datasets. Pearson correlations (*r*) of cAge predictions with reported age were calculated along with the root mean square error (RMSE) and median absolute error (MAE).

#### Log(age)

In addition to training on chronological age, we also trained models on the natural logarithm of chronological age, log(age), using the same LOCO framework as described above. The age of our test samples was predicted using the model trained on chronological age and, if the value returned was 20 years or younger, a new prediction was obtained making use of the model trained on log(age).

#### Feature pre-selection

Several studies have highlighted the benefits of feature pre-selection for elastic net [36, 37]. Here, we performed preliminary analyses, including differently sized subsets of CpG sites as features in elastic net. After filtering for CpGs present across all datasets (374,791), we considered sites that were epigenome-wide significant at *p* < 3.6 × 10^−8^ and then ranked CpGs in ascending order of *p*-value (most significant ranked first), before defining subsets of varying sizes (from 1000 to 300,000 CpGs). For the purpose of selecting an optimal number of pre-selected CpGs, we performed a screening using Generation Scotland as our training cohort and GSE40279 (one of the largest external datasets with a wide age range) as our test set. Our analyses showed that the 10,000 most significant loci (age—CpG associations) yielded the test set predictions with the highest *r* and lowest RMSE (see Results). In addition to these sites, subsets of CpGs with a significant quadratic relationship to age were explored, with subset sizes varying from 100 to 20,000. These features were included in training as CpG^2^ beta values and, when not already present in the model, in their linear form as well. In addition to the top 10,000 age-associated CpGs, the top 300 quadratic sites from our EWAS yielded the best performing model (see Results). This final list of features was then used as input for the LOCO framework described above. The final models, trained on all datasets, selected a *λ* of 0.0308 for the model trained on age and a *λ* of 0.0006 for the model trained on log(age).

#### Comparison to ZhangAge, HannumAge, and HorvathAge

Our final cAge predictor (trained on all 11 datasets in Table 1) and those by Zhang et al. (ZhangAge) [5], Hannum et al. (HannumAge) [4], and Horvath (HorvathAge) [3] were projected onto the GSE55763 dataset to compare their performance in an independent test set. External clock predictions were calculated using the methylCIPHER R package [38] (https://github.com/MorganLevineLab/methylCIPHER).

### Prediction of time to all-cause mortality as a proxy for biological age

#### Training in Generation Scotland

To train a bAge predictor, component scores for GrimAge were estimated for all Generation Scotland samples via Horvath’s online calculator [17] (http://dnamage.genetics.ucla.edu/new). These included EpiScores of smoking and seven proteins—DNAm ADM, DNAm B2M, DNAm cystatin C, DNAm GDF15, DNAm leptin, DNAm PAI1, and DNAm TIMP1. Each variable was then standardised to have a mean of zero and variance of one. We also considered DNAm EpiScores for 109 proteins as described by Gadd et al. [14]. The 109 EpiScores were projected into Generation Scotland via the MethylDetectR [39] Shiny App (https://shiny.igmm.ed.ac.uk/MethylDetectR/) before being standardised to have a mean of zero and variance of one.

This resulted in 116 protein EpiScores, a smoking EpiScore, plus chronological age and sex as features for an elastic net Cox PH model (R package *glmnet* version 4.1.4), using time to all-cause mortality or censoring as outcome. A 20-fold CV was performed (with approximately 1000 individuals per fold), with individuals from the same Generation Scotland technical batch (see Additional file 1) included in the same fold, and with Harrell’s *C* index used to identify the optimal *λ* value (0.0025).

#### Testing in LBC, FHS, and WHI

We defined bAge as the weighted linear combination of covariates selected by our Cox PH elastic net model (see Results). These estimates were then scaled and returned as a predictor with mean of zero and variance of one, for each dataset. A bAgeAccel estimate was also calculated, which is the residual of bAge regressed on chronological age to obtain measure of accelerated epigenetic ageing.

After regressing on age, we assessed the association between our bAge clock, as well as GrimAge, PhenoAge, and DunedinPACE, and time to all-cause mortality in LBC1921 and LBC1936. GrimAge and PhenoAge were calculated using Horvath’s online calculator [17], whilst DunedinPACE was calculated via the DunedinPACE R package [16] (https://github.com/danbelsky/DunedinPACE). Cox PH models, adjusting for age and sex, were used to evaluate associations between the clocks and all-cause mortality. Further, Cox PH models treating GrimAge, PhenoAge, and DunedinPACE (in turn) as a covariate in addition to our bAge clock were run to assess our predictor’s independent association with mortality.

Finally, associations with time to all-cause mortality in four additional external datasets (FHS, and the WHI studies for White, Black, and Hispanic ancestries) were assessed for GrimAge and bAge, the clocks with the largest associations in the LBC cohorts (Table 2).

We examined Schoenfeld residuals in the LBC1921 and LBC1936 Cox PH models that included age, sex, and our bAge clock as covariates to check the proportional hazards assumption at both global and variable-specific levels using the *cox.zph* function from the R *survival* package (version 3.3.1).

#### CpG-based predictor of mortality

We also investigated a direct CpG predictor for time to all-cause mortality (methods and results described in Additional file 2). This predictor was found to have weaker associations with time to all-cause mortality in the LBC cohorts than the aforementioned bAge estimate, both when training just on CpGs as well as when considering both CpGs and EpiScores as training features.

### Enrichment analyses

Gene set enrichment analyses were performed using the Functional Mapping and Annotation (FUMA) GENE2FUNC tool [40], which employs a hypergeometric test. Background genes employed included all unique genes tagged by CpGs in the EPIC array. A false discovery rate (FDR) *p*-value threshold was set at 0.05, and the minimum number of overlapping genes within gene sets was set to 2. These analyses did not explicitly account for Illumina chip biases relating to how CpGs are annotated to genes [41], which may have influenced our results.

## Results

### Epigenome-wide association study of chronological age

EWAS of cAge were performed in the Generation Scotland cohort, resulting in 99,832 linear and 137,915 quadratic CpG associations that were epigenome-wide significant (*p* < 3.6 × 10^−8^, Additional file 3: Figure S1, Additional file 4: Table S1 and S2, see Methods). These mapped to 17,339 and 19,432 unique genes, respectively. There were 48,312 CpGs with both a significant linear and quadratic association.

The most significant linear associations included cg16867657 and cg24724428 (*ELOVL2*), cg08097417 (*KLF14*), and cg12841266 (*LHFPL4)*, all *p* < 1.0 × 10^−300^, (Additional file 3: Figure S2, Additional file 4: Table S1). Around half of the CpGs with a significant linear association (51,213/99,832, 51.3%) showed a positive association between DNAm and age. The most significant quadratic associations were cg11084334 (*LHFPL4*, *p* = 6.5 × 10^−206^), cg15996534 (*LOC134466*, *p* = 8.7 × 10^−194^), and cg23527621 (*ECE2* and *CAMK2N2*, *p* = 1.0 × 10^−190^, Additional file 3: Figure S3, Additional file 4: Table S2).

The univariate associations between all 752,722 CpGs and cAge in a subset of 4450 unrelated participants (DNAm arrays processed together in a single experiment) from Generation Scotland can be visualised via an online ShinyApp, MethylBrowsR (https://shiny.igmm.ed.ac.uk/MethylBrowsR/).

### Epigenome-wide association study of time to all-cause mortality

To identify individual CpG loci associated with survival, we performed an EWAS on time to all-cause mortality in Generation Scotland (*N*_deaths_ = 1214; see Methods). This analysis identified 1182 epigenome-wide significant associations (*p* < 3.6 × 10^−8^, Additional file 3: Figure S4), which mapped to 704 unique genes. For around a third (418/1182 = 35.4%) of these CpGs, DNAm was associated with a decreased survival time (HR > 1). The lead findings included CpGs mapping to smoking-related loci [10, 42–46] such as cg05575921 (*AHRR*, *p* = 3 × 10^−57^), cg03636183 (*F2RL3*, *p* = 6.8 × 10^−44^), cg19859270 (*GPR15*, *p* = 1.1 × 10^−33^), cg17739917 (*RARA*, *p* = 1.9 × 10^−33^), cg14391737 (*PRSS23, p* = 5.6 × 10^−33^), cg09935388 (*GFI1, p* = 3.3 × 10^−31^), and cg25845814 (*ELMSAN1/MIR4505*, *p* = 1.3 × 10^−30^) (Additional file 4: Table S3). Amongst the top 50 associations, only one probe has not been previously linked to smoking (assessed via a lookup of findings from the EWAS catalog [47]), cg03546163. This probe maps to *FKBP5*, a gene whose methylation is involved in the regulation of the stress response and which has been linked to increased cardiometabolic risk through accelerated ageing [48]. All associations, except that for cg24364998, remained statistically significant after adjusting for relatedness in the Generation Scotland cohort (see Methods, Additional file 4: Table S4).

There was a high correlation of the *Z*-score effect sizes across the 200 sites that overlapped between our study and the 257 epigenome-wide significant findings from a recent large (*N* = 12,300, *N*_deaths_ = 2561) meta-analysis of all-cause mortality [49] (*r* = 0.58, Additional file 3: Figure S5). Despite differences in covariate adjustments, all 200 sites were significant at a nominal *p* < 0.05 threshold, and 25 were epigenome-wide significant at *p* < 3.6 × 10^−8^.

A gene-set enrichment analysis considering genes to which epigenome-wide significant CpGs mapped returned 198 significantly enriched (FDR *p* < 0.05) GO biological processes (see Methods, full FUMA gene-set enrichment results in Additional file 4: Table S5). The most significantly enriched GO terms included processes relating to neurogenesis/neuron differentiation and development, positive immune system regulation and development, cell motility and organisation, and regulation of protein modification/phosphorylation. Other significantly enriched sets included sites bound by FOXP3*,* ETS2, and the PML-RARA fusion protein.

### Prediction of chronological age

Epigenetic clocks for cAge were created using elastic net penalised regression in a LOCO framework (total of 10 models), with a final cAge clock trained on all data (see Methods, Fig. 2, Additional file 2). In our screening step, after iterating through combinations of CpG and CpG^2^ terms (ranked by EWAS *p*-value), the best-performing model considered the top 10,000 CpG and top 300 CpG^2^ sites from the EWAS as potentially informative features (see Methods, Additional file 3: Figure S6 and S7, Additional file 4: Table S6 and S7). Both age and log(age) were considered as outcomes, with the latter showing better prediction results in younger individuals, reflecting the importance of considering non-linear DNAm-age associations in cAge prediction (see Methods, Additional file 2). As a result, if the initial cAge prediction was < 20 years, that individual’s predicted age was re-estimated using weights from the log(age) model.

**Fig. 3:**
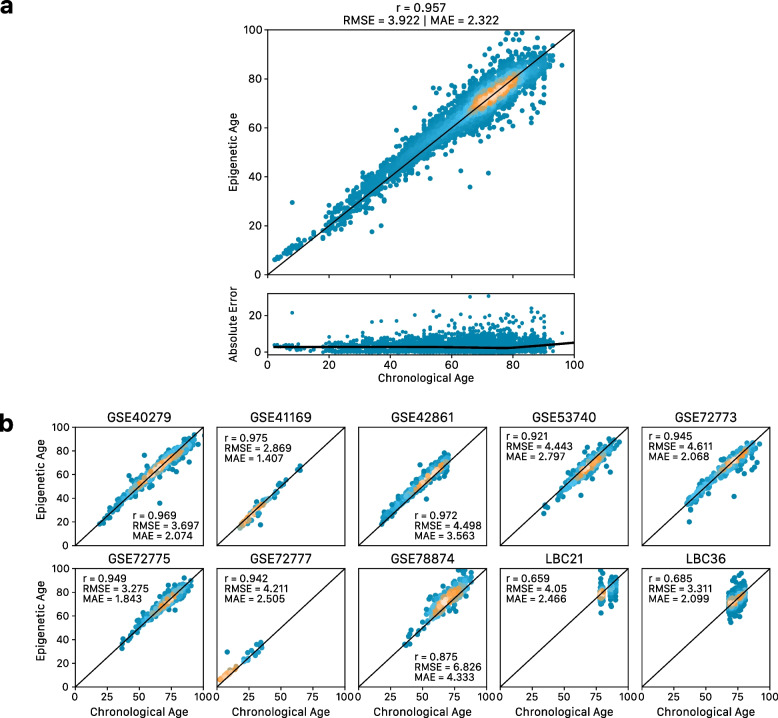
Performance of cAge LOCO framework (one cAge model per external cohort), **a** across all 10 datasets considered, and **b** per cohort. Performance metrics shown include Pearson correlation (*r*), root mean squared error (RMSE), and median absolute error (MAE). Metrics also included in Table 1

The combined LOCO prediction results (one cAge model per external cohort) showed a strong correlation with cAge (*r* = 0.96, Fig. 3, Additional file 3: Figure S8, Table 1) and a MAE of 2.3 years. Furthermore, 24% of individuals were classified to within 1 year of their chronological age. The cohort with the largest prediction errors was GSE78874, in which DNAm was measured in saliva instead of blood.

The final cAge predictor (trained in all 11 cohorts) with the lowest mean cross-validated error identified 2330 features (2274 linear and 56 quadratic) as most predictive of age, and 1986 features (1931 linear and 55 quadratic) as most predictive of log(age). The weights for the age model are presented in Additional file 4: Table S8, and for the log(age) model in Additional file 4: Table S9.

**Fig. 4:**
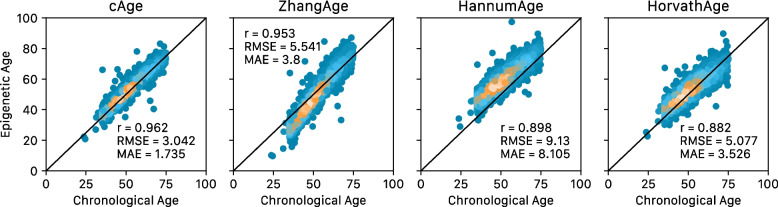
cAge predictor performance in the GSE55763 dataset, compared to ZhangAge, HannumAge, and HorvathAge. Performance metrics shown include Pearson correlation (*r*), root mean squared error (RMSE), and median absolute error (MAE)

Considering a large external cohort (*N* = 2711), our cAge predictor (*r* = 0.96, RMSE = 3.04, MAE = 1.74) outperformed ZhangAge (*r* = 0.95, RMSE = 5.54, MAE = 3.8), HorvathAge (*r* = 0.90, RMSE = 9.1, MAE = 8.11), and HannumAge (*r* = 0.88, RMSE = 5.08, MAE = 3.53, Fig. 4).

### Prediction of time to all-cause mortality as a proxy for biological age

**Fig. 5:**
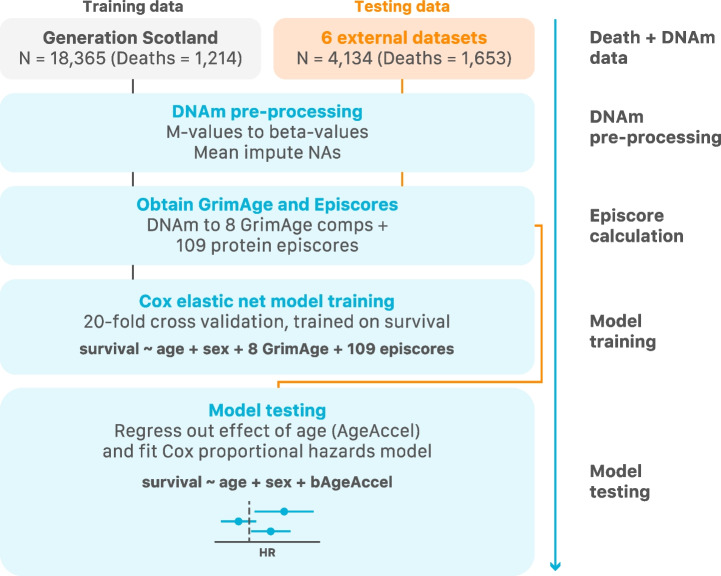
Flowchart for the creation of the bAge predictor. First, DNAm data originating from Generation Scotland and six external datasets was pre-processed. GrimAge components and 109 protein EpiScores were generated within each cohort. A Cox PH elastic net regression model of time to all-cause mortality (with 20-fold CV) was trained in Generation Scotland with the GrimAge components and EpiScores as possible features. The model that maximised Harrell’s *C* index was tested on the six external datasets

In an effort to improve the prediction of bAge, an elastic net Cox model was trained on time to all-cause mortality in Generation Scotland (*N*_total_ = 18,365, *N*_deaths_ = 1214; see Methods). The GrimAge components (age, sex, and EpiScores for smoking and 7 plasma proteins) and Gadd et al.’s 109 protein EpiScores [14] were considered as potentially-informative features (Fig. 5).

The elastic net Cox PH model identified a weighted sum of 35 features as most predictive of time to all-cause mortality in Generation Scotland. These included age and the GrimAge smoking EpiScore, along with 5/7 protein EpiScores from GrimAge (B2M, cystatin C, GDF15, PAI1, and TIMP1), and 28/109 protein EpiScores from Gadd et al. [14]. Amongst these were EpiScores for C-reactive protein (CRP), the growth hormone receptor (GHR) protein, and numerous cytokines (CCL11, CCL23, CCL18, CXCL10, CXCL9, CXCL11, and HGF). The weights for the linear predictor are presented in Additional file 4: Table S10.

**Fig. 6:**
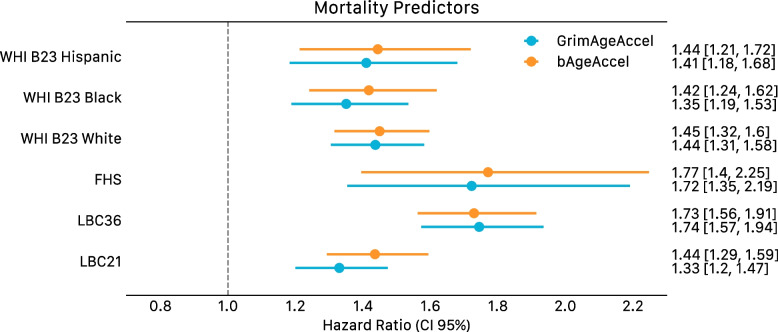
Forest plots of bAge/GrimAge predictors, applied to time to all-cause mortality in LBC1921, LBC1936, FHS, and WHI. Predictors regressed on age. Hazard ratios are presented per standard deviation of the GrimAgeAccel and bAgeAccel variables, along with 95% confidence intervals. Cox models are adjusted for age at DNAm sampling and sex

Our bAge predictor was regressed on age to obtain a measure of epigenetic age acceleration (bAgeAccel). The epigenetic age acceleration residuals showed significant associations with time to all-cause mortality across six test datasets of differing ancestries (Table 2, Additional file 4: Table S11, Fig. 6).

We assessed our predictor’s association with time to all-cause mortality in comparison to three other epigenetic clocks: GrimAge, PhenoAge, and DunedinPACE (age acceleration residuals after regressing the clock estimates on age) in the LBC1921 and LBC1936 cohorts (see Methods). Our bAge predictor showed stronger associations (in magnitude and statistical significance) with time to all-cause mortality than DunedinPACE and PhenoAge and similar performance to GrimAge (Additional file 3: Figure S9, Additional file 4: Table S12). Our bAge predictor’s association with time to all-cause mortality remained significant (*p* < 0.05) after adjusting for GrimAge, PhenoAge, and DunedinPACE as covariates in three separate models (Additional file 4: Table S13). Considering all six test datasets, the bAge measure showed slightly stronger associations than GrimAge in fixed effects meta-analyses (HR and 95% confidence interval per SD difference of GrimAgeAccel and bAgeAccel: HR = 1.47 [1.40, 1.54] with *p* = 1.08 × 10^−52^, and HR = 1.52 [1.44, 1.59] with *p* = 2.20 × 10^−60^, respectively (Table 2, Additional file 4: Table S11, Fig. 6).

Schoenfeld residual analyses highlighted violations to the proportional hazards assumption at global and variable specific levels for LBC1921 and LBC1936. However, re-running the analysis with different TTE censoring (thresholding at each possible integer year of follow-up) showed minimal differences in the bAgeAccel-survival HRs between models that did not violate the assumption and those that did (see Methods, Additional file 4: Table S14).

## Discussion

Accurate predictors of cAge and bAge have major implications for biomedical science and healthcare through risk prediction and preventative medicine. Here, we present improved DNAm-based predictors of age and lifespan.

Epigenetic cAge prediction is expected to reach near-perfect estimates as sample sizes grow [5]. Making use of Generation Scotland, a very large single-cohort DNAm resource, we derived a cAge predictor with a MAE of 1.7 years, tested in over 2000 external samples (Fig. 4). Our predictor has potential forensic applications, although ethical caveats exist [8]. In addition, despite the high correlations and low RMSE and MAE estimates at the population level, there are still several individuals with inaccurate predictions (e.g. > 20 years between predicted and actual age, Fig. 3), though this could also reflect sample mix-ups or data entry errors.

cAge prediction was improved when accounting for non-linear relationships between DNAm and age (Additional file 2, Additional file 3: Figure S7). Whilst generally understudied, non-linear patterns have been found at numerous CpG sites, where DNAm is found to increase rapidly in early ages and stabilise in adulthood, potentially reflecting developmental processes [50]. Similarly, stable DNAm levels followed by rapid methylation/demethylation have also been described in later life [51], which could offer insight into ageing-specific processes. Given the number of samples from individuals aged 20 or under in the training of our predictor (*N* = 574/24,674 = 2.4%), we may not have captured the full extent of DNAm-based ageing patterns in the younger population. Future studies could also consider sex-specific models, as diverging non-linear patterns between males and females have been shown previously [52]. Interactions between CpGs along with higher order polynomial terms and spline-based models might better capture some of these non-linear changes.

The development of the cAge predictor highlighted the advantages of feature pre-selection ahead of penalised elastic net regression. Compared to a model with all possible features in the training set (*r* = 0.93, RMSE = 5.25, MAE = 3.43, see Additional file 2), pre-selection greatly improved performance (*r* = 0.96, RMSE = 3.92, MAE = 2.32, Fig. 3). Several DNAm studies of age and age-related phenotypes have used pre-selection methods (e.g. filtering by magnitude of correlation or strength of association) instead of, or in addition to elastic net [53–60]. Whereas the feature pre-selection here required arbitrary decisions on thresholds, other studies have found that feature reduction via PCA optimises DNAm predictors [36, 37].

Feature pre-selection may have aided cAge predictions by screening out CpGs with low intra-sample variability due to technical variance [61, 62]. One previous study [37] observed that CpGs with stronger cAge associations were more reliable. A limitation of our approach to feature pre-selection was that it was biased towards the Generation Scotland cohort in which the age EWAS were conducted. We also note that pre-selection introduces statistical challenges associated with post-selection inference [63]. Furthermore, our penalised regression modelling strategy for cAge only incorporated additive effects. Non-additive tree ensemble methods and other machine learning frameworks may improve predictions further [64]. Finally, as our predictor has been mainly trained and tested on blood data, it may not generalise to other tissues.

Whilst a single DNAm predictor of cAge is of interest, the selected CpG features are unlikely to identify all epigenome-wide patterns related to ageing. Our EWAS of chronological age identified 99,832 linear and 137,915 quadratic CpG-age associations. The sample size was more than double that of the largest study reported on the EWAS Catalog [47]—our previous Generation Scotland analysis [65]. In addition to refining our previously described DNAm-age linear associations, we have extended previous small-scale approaches to highlight non-linear patterns [51, 52]. As shown here, these findings can aid the predictive performance of epigenetic clocks and may additionally improve our understanding of epigenetic changes during development and ageing-related decline in later life.

Recent work has shifted focus from the prediction of cAge to bAge, with more expansive clinical applications. Our new bAge predictor of all-cause mortality had a greater effect size and was more statistically significant than GrimAge in the external test set meta-analysis. GrimAge is already being used as an end-point for clinical trials [66] and studies of rejuvenation [67, 68]. Our bAge predictor includes five of the seven original GrimAge EpiScores, with ADM and leptin not being selected as features. In addition, it includes 28 protein EpiScores from Gadd et al. [14]. Amongst the additional protein EpiScores selected by our predictor were those for CRP and numerous cytokines, which reflect inflammation and predict overall and cardiovascular mortality [69–71]. Chronic inflammation can lead to several diseases, including cardiovascular disease, and exacerbates the ageing process [72, 73]. In addition, the growth hormone receptor (GHR) protein EpiScore was selected; both the receptor and its corresponding protein have been linked to longevity in mouse models [74–78]. Twenty-five of the 28 of the selected EpiScores from Gadd et al. [14] have been associated to multiple diseases, including diabetes, chronic obstructive pulmonary disease, ischaemic heart disease, lung cancer, Alzheimer’s, rheumatoid arthritis, stroke, and depression (Additional file 4: Table S10). As sample sizes for cause-specific mortality outcomes increase, a more granular suite of lifespan predictors can be developed. Future studies may also consider the cost implications of profiling thousands of CpGs against the potential improvements in health-span and savings from delaying or preventing disease.

Whereas the cAge predictor is directly applicable and interpretable for a new individual, bAge estimates are relative to the values of other participants in the testing dataset, given the within-cohort scaling of the input features prior to projection. Reporting findings per SD of bAgeAccel will therefore help to facilitate cross-cohort comparisons. Future work for these (and all) DNAm array-based predictors should consider the limitations of signatures that lack absolute thresholds/cut-points for risk prediction in a new individual selected at random from the population.

A total of 1182 epigenome-wide significant associations were identified in our EWAS of all-cause mortality. The most significant probes mapped to genes previously associated with smoking, such as *AHRR*, *F2RL3*, and *GPR15* [79]. Hypomethylation at probes nearby these genes has been previously linked to increased mortality risk, be that all-cause or disease specific (e.g. cancer or cardiovascular-related mortality) [29, 42, 80, 81]. There was moderate agreement (correlation of 0.58 between *Z*-scores) between our findings and the significant results from a previous EWAS meta-analysis of survival. However, different covariates and ancestries were considered across these studies. An enrichment analysis highlighted links to neurodevelopment and immune regulation, as well as to sites bound by FOXP3*,* ETS2, and the PML-RARA fusion protein. FOXP3 is a transcriptional regulator involved in the development and inhibitory function of regulatory T cells [82]. ETS2 and PML-RARA are a protooncogene and a protein resulting from a chromosomal translocation that generates an oncofusion protein, respectively, having both been linked to acute myeloid leukemia [83, 84]. This finding may be influenced by the large number of cancer-related deaths in Generation Scotland (*N* = 509). Further work is needed to disentangle the role of methylation/demethylation at these sites with survival, including the fitting of models with more complete sets of comorbidities, risk, and lifestyle factors. Future EWAS on specific mortality causes will highlight mechanisms underlying age- and disease-related decline.

Importantly, the majority of Generation Scotland participants are of White British ancestry, meaning analyses could present biases towards this population. Whilst our cAge predictor, which was trained on Generation Scotland and external cohorts of multiple ancestries (White, Hispanic, South Asian, East Asian), showed similar accuracy across all testing datasets (Fig. 3b), the magnitude of the survival effect size for bAge was slightly reduced (though still statistically significant) when considering African American and Hispanic ancestry samples, as opposed to European American samples in the WHI cohort (Fig. 6, Table 2). Additionally, our EWASs of survival and age were conducted only using Generation Scotland data. In this context, large multi-ancestry and multi-omic cohorts are needed.

## Conclusions

The integration of multiple large datasets and new approaches to feature selection has facilitated improvements to the blood-based epigenetic prediction of biological and chronological age. The inclusion of multiple protein EpiScore features and consideration of quadratic DNAm effects may also be relevant for other EWAS and prediction studies. Together, this can improve our biological understanding of complex traits and the prediction of adverse health outcomes.

## Supplementary Information

**Additional file 1. **Dataset descriptions for Generation Scotland, LBC1921 and LBC1936, GEO datasets, FHS, and WHI. (PDF 88Kb).

**Additional file 2. **Additional cAge and bAge predictor analyses that informed the decisions made in the creation of the latter as described in this manuscript. (PDF 3.3Mb).

**Additional file 3. Figure S1.** Manhattan plots of linear and quadratic age EWAS in Generation Scotland. **Figure S2.** Scatterplots of top 15 associations from age ~ CpG EWAS. **Figure S3.** Scatterplots of top 15 associations from the age ~ CpG + CpG^2^ EWAS. **Figure S4.** Manhattan plot of all-cause mortality EWAS. **Figure S5.** Comparison of Z-values for 200 overlapping epigenome-wide significant CpG-mortality associations reported by Colicino et al., and those considered in the present study. **Figure S6.** Prediction metrics for GSE40279, as a function of CpGs included in training of a cAge predictor, trained using Generation Scotland data. **Figure S7.** Prediction metrics for GSE40279, as a function of CpG^2^s included in training of a cAge predictor trained using Generation Scotland data, in addition to top 10K age-associated CpGs. **Figure S8.** cAge predictor median absolute error (MAE) +/- 1.96 its standard deviation on 10 external testing datasets, as a function of age, in 10 year intervals. **Figure S9.** Forest plots of bAge, GrimAge, PhenoAge, and DunedinPACE predictors, applied to all-cause mortality in LBC1921 and LBC1936. (PDF 12.2Mb).

**Additional file 4. Table S1.** Top 10,000 epigenome-wide significant associations between CpG and age. **Table S2.** Top 10,000 epigenome-wide significant associations between CpG^2 and age. **Table S3.** Epigenome-wide significant CpG associations with all-cause mortality. **Table S4.** Epigenome-wide significant CpG associations with all-cause mortality, replicated using the coxme R package to account for relatedness. **Table S5.** Gene-set enrichment results for mortality EWAS hits, as returned by FUMA. **Table S6.** Model performance as a function of CpG subset size input into elastic net. **Table S7.** Model performance as a function of CpG^2^ subset size input into elastic net, in addition to top 10K linear CpG-age associated sites. **Table S8.** Components of cAge predictor, along with their respective weights. **Table S9.** Components of log(cAge) predictor, along with their respective weights. **Table S10.** bAge predictor components, along with their weights. **Table S11.** bAgeAccel and GrimAgeAccel associations to mortality across 6 datasets. **Table S12.** bAgeAccel, GrimAgeAccel, PhenoAgeAccel, and DunedinPACEAccel associations to mortality across 2 datasets. **Table S13.** bAgeAccel associations to mortality across 2 datasets, including another biological age clock as a covariate (GrimAgeAccel, PhenoAgeAccel, or DunedinPACEAccel). **Table S14.** Schoenfeld residuals and HR as a function of TTE cut-off in bAge prediction. (XLSX 5.3Mb).

## Data Availability

According to the terms of consent for Generation Scotland participants, access to data must be reviewed by the Generation Scotland Access Committee. Applications should be made to access@generationscotland.org. Lothian Birth Cohort data are available on request from the Lothian Birth Cohort Study, University of Edinburgh (https://www.ed.ac.uk/lothian-birth-cohorts/data-access-collaboration). Lothian Birth Cohort data are not publicly available due to them containing information that could compromise participant consent and confidentiality. All custom R (version 4.0.3), Python (version 3.9.7), and bash code is available with open access at the following GitHub repository [85]: https://github.com/elenabernabeu/cage_bage EWAS summary statistics are available on Edinburgh DataShare, along with mean and SD of CpG *M*-values and *F*-test results [86]: https://datashare.ed.ac.uk/handle/10283/4781 (https://doi.org/10.7488/ds/3792). cAge predictions can be obtained using MethylDetectR (https://shiny.igmm.ed.ac.uk/MethylDetectR/) or via a standalone script: https://github.com/elenabernabeu/cage_bage/tree/main/cage_predictor As the CpG weights for the GrimAge components are not publicly available, bAge predictions first require users to generate GrimAge estimates from the following online calculator (http://dnamage.genetics.ucla.edu/new). bAge can then be estimated via the following standalone script: https://github.com/elenabernabeu/cage_bage/tree/main/bage_predictor Visualization of CpG-age relationships can be viewed using MethylBrowsR: https://shiny.igmm.ed.ac.uk/MethylBrowsR/
