# Supplementary material for: Refining epigenetic prediction of chronological and biological age
Source: Genome Med. 2023 Feb 28;15:12. doi: 10.1186/s13073-023-01161-y (PMC9976489; doi:10.1186/s13073-023-01161-y)
Supplement: Supplementary file 1 — Additional file 1. Dataset descriptions for Generation Scotland, LBC1921 and LBC1936, GEO datasets, FHS, and WHI. (PDF 88Kb). [file 13073_2023_1161_MOESM1_ESM.pdf]

Dataset Description

Generation Scotland

DNA methylation in blood was quantified for 18,413 Generation Scotland participants across three separate sets (N_{Set1} = 5,087, N_{Set2} = 4,450, N_{Set3} = 8,876) using the Illumina MethylationEPIC (850K) array. Individuals in Set 1 included a mixture of related and unrelated individuals. Set 2 comprised individuals unrelated to each other and also to those in Set 1. Set 3 contained a mix of related individuals – both to each other and to those in Sets 1 and 2 – and included all remaining samples available for analysis. Methylation data was processed across 121 experimental batches ($N_{Batches.\ Set1}$ = 31, $N_{Batches,\ Set2}$ = 30, $N_{Batches,\ Set3}$ = 60).

Quality control details have been reported previously[1,2]. Briefly, probes were removed based on (i) outliers from visual inspection of the log median intensity of the methylated versus unmethylated signal per array, (ii) a bead count < 3 in more than 5% of samples, (iii) ≥ 5% of samples having a detection *p*-value > 0.05, (iv) if they pertained to the sex chromosomes, (v) if they overlapped with SNPs, and/or (vi) if present in potential cross-hybridizing locations[3]. Samples were removed (i) if there was a mismatch between their predicted sex and recorded sex, (ii) if ≥ 1% of CpGs had a detection *p*-value > 0.05, (iii) if sample was not blood-based, and/or (iv) if participant responded "yes" to all self-reported diseases in questionnaires. Dasen normalisation[4] was carried out per set (for cAge training) or across all individuals (for EWAS). A total of 752,722 CpGs remained after QC. To maximise the generalisability of the predictors across different versions of Illumina arrays, we subset the content to the intersection of sites on the EPIC and 450K arrays, as well as to those present across all cohorts considered in the study (**Table 1**), totalling 374,791 CpGs. Missing values were mean imputed.

External datasets

For the training and testing of our cAge predictor, we considered DNA methylation for a total of 6,261 external samples, from eight publicly available datasets from the Gene Expression Omnibus (GEO) resource and repeated measures (up to four time points) from two cohorts of blood-based DNAm, the Lothian Birth Cohorts (LBC) of 1936 and 1921 (**Table 1**)[5–11]. In addition, the GEO GSE55763[12,13] dataset (2,711 samples from 2,664 individuals) was used to assess cAge clock performance against existing clocks.

For the testing of our bAge predictor, the baseline samples from the LBC cohorts, along with the Framingham Heart Study (FHS) and the Women's Health Initiative (WHI) study, were used (**Table 2**).

Lothian Birth Cohorts

LBC1921 and LBC1936 are longitudinal studies of ageing on individuals born in 1921 and 1936, respectively[5]. Study participants completed the Scottish Mental Surveys of 1932 and 1947 at approximately age 11 years old and were living in the Lothian area of Scotland at the time of recruitment in later life. Blood samples considered here were collected at around age 79 for LBC1921, and at around age 70 for LBC1936. DNA methylation was quantified using the Illumina HumanMethylation450K array, for a total of 692 (up to 3 repeated measurements from 469 individuals) and 2,796 (up to 4 repeated measurements from 1,043 individuals) samples from LBC1921 and LBC1936 respectively. Quality control details have been reported previously[14,15]. Briefly, probes were removed (i) if they presented a low (< 95%) detection rate with *p*-value < 0.01, and/or (ii) if they presented inadequate hybridization, bisulfite conversion, nucleotide extension, or staining signal, as assessed by manual inspection. Samples were removed (i) if they presented a low call rate (<450,000 probes detected at *p*-value < 0.01) and/or (ii) if predicted sex did not match

reported sex. Finally, as stated previously, probes were filtered down to the 374,791 common across all datasets (**Table 1**). Missing values were mean imputed.

A total of 421 and 895 samples from LBC1921 and LBC1936 respectively, corresponding to the first wave of each study (at mean ages of 79 and 70 years at time of sampling, respectively), were used in our bAge analysis (**Table 2**). All-cause mortality was assessed via linkage to the National Health Service Central Register, provided by the National Records of Scotland. The data used here are correct as of January, 2022, with a total of 421 and 367 deaths in LBC1921 and LBC1936 respectively.

Gene Expression Omnibus (GEO) datasets

DNAm and age information for 2,773 individuals from a total of 8 datasets was downloaded from the public domain (GEO). DNAm was quantified with Illumina's HumanMethylation450K chip. Quality control information can be found in the publications highlighted in **Table 1**. CpGs were filtered down to the 374,791 common across all datasets with any missing values replaced via mean imputation.

Data from the GEO GSE55763 dataset[12,13], with DNAm and age information for 2,664 individuals (2,711 samples), was also downloaded to assess a final cAge clock (trained using all cohorts listed in **Table 1**). This predictor was compared to existing clocks[9,15,16], none of which utilised data from the test set for their development.

Framingham Heart Study (FHS)

The FHS cohort is a large-scale longitudinal study started in 1948, initially investigating the common factors of characteristics that contribute to cardiovascular disease (CVD)[17]. The study at first enrolled participants living in the town of Framingham, Massachusetts, who were free of overt symptoms of CVD, heart attack or stroke at enrolment. In 1971, the study established the FHS Offspring Cohort to enroll a second generation of the original participants' adult children and their spouses for conducting similar examinations[18]. Participants from the FHS Offspring Cohort were eligible for our study if they attended both the seventh and eighth examination cycles and consented to having their molecular data used for study. We used data pertaining to a total of 711 individuals which had not been used in the training of GrimAge, and for which DNAm data and death records were available. Peripheral blood samples were obtained on the eight examination cycle, and DNAm data was measured using the Illumina Infinium HumanMethylation450 array, with QC details are described elsewhere[19]. Deaths recorded are accurate as of 1st January 2013, with a total of 100 recorded.

Women's Health Initiative (WHI)

The WHI study enrolled postmenopausal women aged 50-79 years into the clinical trials (CT) or observational study (OS) cohorts between 1993 and 1998. We included 2,107 women from "Broad Agency Award 23" (WHI BA23). WHI BA23 focuses on identifying miRNA and genomic biomarkers of coronary heart disease (CHD), integrating the biomarkers into diagnostic and prognostic predictors of CHD and other related phenotypes. This cohort is divided into three datasets, pertaining to three different ancestries: White (European American), Black (African American), and Hispanic, with 998, 676, and 433 participants respectively. Blood-derived DNAm data was available for participants. DNAm data was measured using the Illumina Infinium HumanMethylation450 array, QC details described elsewhere[19]. Deaths recorded are accurate as March 1st, 2017, with a total of 418, 229, and 118 recorded for White, Black, and Hispanic ancestries respectively.

References

1. McCartney, D. L. *et al.* Investigating the relationship between DNA methylation age acceleration and risk factors for Alzheimer's disease. *Alzheimer's Dement. Diagnosis, Assess. Dis. Monit.* **10**, 429–437 (2018).
2. McCartney, D. L. *et al.* An epigenome-wide association study of sex-specific chronological ageing. *Genome Med.* **12**, 1–11 (2019).
3. McCartney, D. L. *et al.* Identification of polymorphic and off-target probe binding sites on the Illumina Infinium MethylationEPIC BeadChip. *Genomics Data* **9**, 22 (2016).
4. Pidsley, R. *et al.* A data-driven approach to preprocessing Illumina 450K methylation array data. *BMC Genomics* **14**, 1–10 (2013).
5. Deary, I. J., Gow, A. J., Pattie, A. & Starr, J. M. Cohort profile: the Lothian Birth Cohorts of 1921 and 1936. *Int. J. Epidemiol.* **41**, 1576–1584 (2012).
6. Taylor, A. M., Pattie, A. & Deary, I. J. Cohort Profile Update: The Lothian Birth Cohorts of 1921 and 1936. *Int. J. Epidemiol.* **47**, 1042–1060 (2018).
7. Horvath, S. *et al.* An epigenetic clock analysis of race/ethnicity, sex, and coronary heart disease. *Genome Biol.* **17**, 1–23 (2016).
8. Horvath, S. *et al.* Aging effects on DNA methylation modules in human brain and blood tissue. *Genome Biol.* **13**, R97 (2012).
9. Hannum, G. *et al.* Genome-wide Methylation Profiles Reveal Quantitative Views of Human Aging Rates. *Mol. Cell* **49**, 359–367 (2013).
10. Liu, Y. *et al.* Epigenome-wide association data implicate DNA methylation as an intermediary of genetic risk in rheumatoid arthritis. *Nat. Biotechnol.* **31**, 142–147 (2013).
11. Li, Y. *et al.* An Epigenetic Signature in Peripheral Blood Associated with the Haplotype on 17q21.31, a Risk Factor for Neurodegenerative Tauopathy. *PLOS Genet.* **10**, e1004211 (2014).
12. Wahl, S. *et al.* Epigenome-wide association study of body mass index, and the adverse outcomes of adiposity. *Nature* **541**, 81–86 (2017).
13. Lehne, B. *et al.* A coherent approach for analysis of the Illumina HumanMethylation450 BeadChip improves data quality and performance in epigenome-wide association studies. *Genome Biol.* **16**, (2015).
14. Marioni, R. E. *et al.* DNA methylation age of blood predicts all-cause mortality in later life. *Genome Biol.* **16**, 1–12 (2015).
15. Zhang, Q. *et al.* Improved precision of epigenetic clock estimates across tissues and its implication for biological ageing. *Genome Med.* **11**, 1–11 (2019).
16. Horvath, S. DNA methylation age of human tissues and cell types. *Genome Biol.* **14**, 1–20 (2013).
17. Dawber, T. R., Meadors, G. F. & Moore, F. E. Epidemiological approaches to heart disease: the Framingham Study. *Am. J. Public Health* **41**, 279–281 (1951).
18. Kannel, W. B., Feinleib, M., Mcnamara, P. M., Garrison, R. J. & Castelli, W. P. An investigation of coronary heart disease in families. The Framingham offspring study. *Am. J. Epidemiol.* **110**, 281–290 (1979).
19. Lu, A. T. *et al.* DNA methylation GrimAge strongly predicts lifespan and healthspan. *Aging (Albany. NY).* **11**, 303–327 (2019).
