# Supplementary material for: Refining epigenetic prediction of chronological and biological age
Source: Genome Med. 2023 Feb 28;15:12. doi: 10.1186/s13073-023-01161-y (PMC9976489; doi:10.1186/s13073-023-01161-y)
Supplement: Supplementary file 2 — Additional file 2. Additional cAge and bAge predictor analyses that informed the decisions made in the creation of the latter as described in this manuscript. (PDF 3.3Mb). [file 13073_2023_1161_MOESM2_ESM.pdf]

# Supplementary Predictor Analyses

This file briefly describes the supplementary analyses and results that informed the creation of chronological age (cAge) and biological age (bAge) predictors, as described in our manuscript "Refining epigenetic prediction of chronological and biological age". For a detailed description of the Methods as well as the data employed, please refer to the main article file.


*cAge predictors* ........ *2*

1. Training in GS, testing in external cohorts, no log(age), no feature pre-selection ........ 3
2. LOCO with no log(age), no feature pre-selection ........ 4
3. LOCO with no log(age), feature pre-selection ........ 5
4. LOCO with log(age), no feature pre-selection ........ 6
5. LOCO with log(age), feature pre-selection ........ 7

*bAge predictors* ........ *8*

1. EWAS based CpG predictor ........ 8
2. EWAS based CpG predictor + EpiScores ........ 10

## cAge predictors

The cAge predictor presented in our manuscript was trained using an elastic net penalised regression framework, with DNA methylation (DNAm) data at CpG sites as training features (total CpGs after QC = 374,791). The bulk of our training data originated from the Generation Scotland cohort (GS, N = 18,413), and we further considered 10 external datasets for both training and testing (N = 6,261), including 2 Lothian Birth Cohorts (LBC) and 8 datasets from the Gene Expression Omnibus (GEO).

To develop the predictor, we (1) used a leave-one-cohort-out (LOCO) framework for training/testing, (2) performed feature pre-selection ahead of elastic net (for both linear and quadratic CpG features), and (3), trained on both linear age as well as log(age) to account for potential non-linear methylation patterns in early life (under 20 years old). Here, we describe how each of these steps/methods influenced our predictor.

## 1. Training in GS, testing in external cohorts, no log(age), no feature pre-selection

The first model fit was trained only on the GS cohort, using the parameters stipulated in the Methods section of the main manuscript. It used as training features DNAm data for all available CpGs (N = 374,791). The resulting predictor was then tested on the 10 external datasets. This predictor had a Pearson correlation (r) of 0.91, a root mean squared error (RMSE) of 7.8 and a median absolute error (MAE) of 5.88 years across all external samples (**Additional file 2: Figure 1**). There were notable differences between predicted and observed values at both ends of the age spectrum.

**a)**

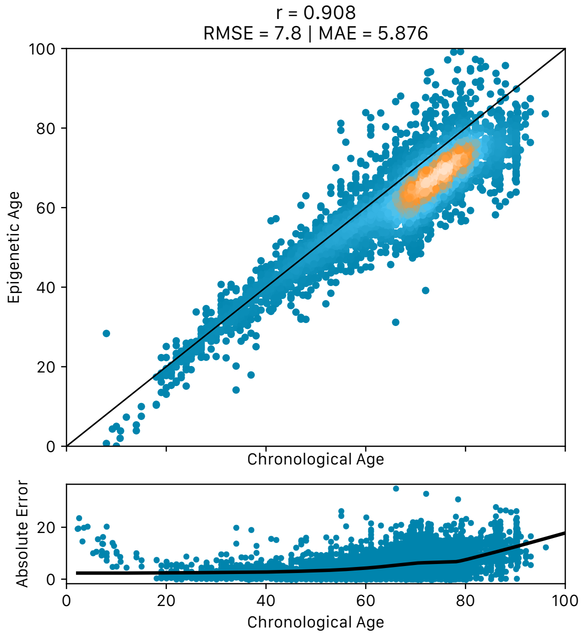


**b)**

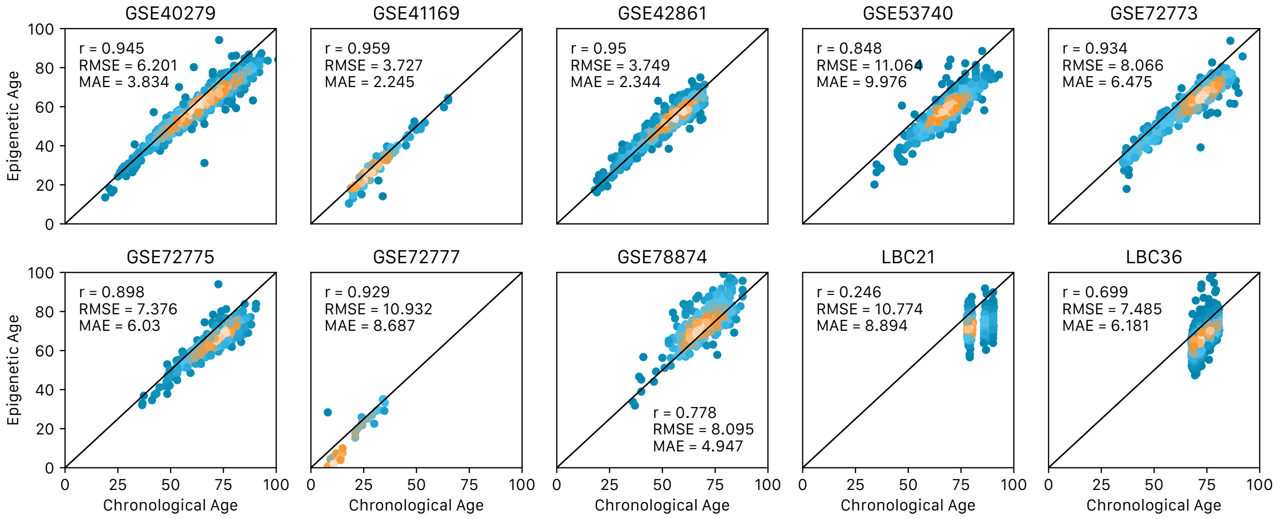


**Additional file 2: Figure 1. cAge predictor (trained on GS, linear age, and with no feature pre-selection) performance on 10 external testing datasets**, (a) across all datasets considered, and (b) per cohort. Performance metrics shown include Pearson correlation (r), root mean squared error (RMSE), and median absolute error (MAE).

## 2. LOCO with no log(age), no feature pre-selection

To avoid creating a predictor that was overfitted to the GS population, we next explored a LOCO approach, by which a model was trained on GS and all but one external dataset, and then tested on the excluded dataset (see Methods in manuscript for more details). Once again, all CpGs available were used in training. The resulting model performed with r = 0.92, RMSE = 5.25, and MAE = 3.41 (**Additional file 2: Figure 2**). Whilst this was an improvement from the first model, notable prediction errors in younger and older individuals were still observed.

**a)**

**b)**

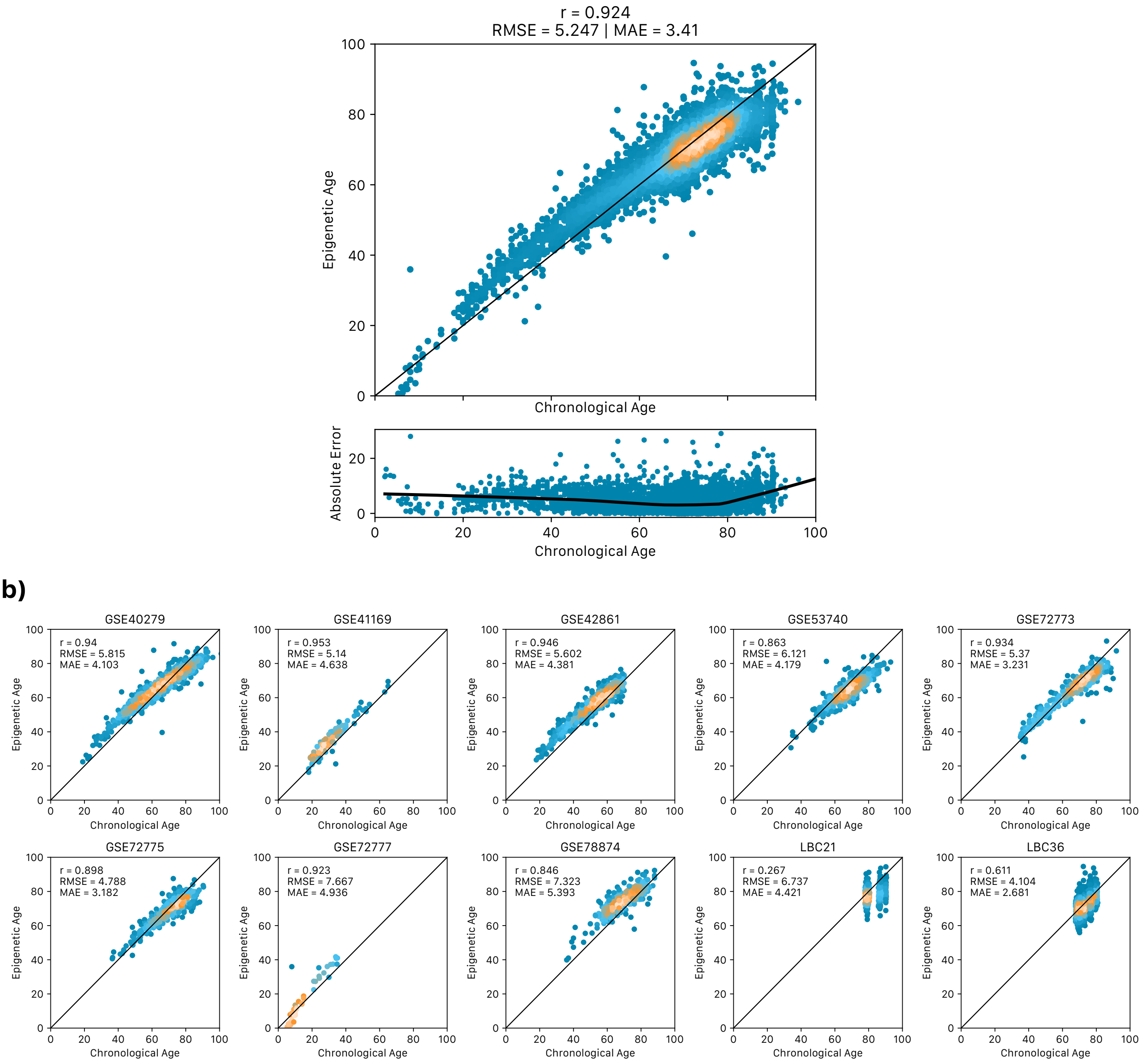


**Additional file 2: Figure 2. cAge predictor (trained using LOCO framework, on linear age, and with no feature pre-selection) performance on 10 external testing datasets**, (a) across all datasets considered, and (b) per cohort. Performance metrics shown include Pearson correlation (r), root mean squared error (RMSE), and median absolute error (MAE).

## 3. LOCO with no log(age), feature pre-selection

Our next approach was informed by the EWAS performed and described in the main manuscript, which considered both linear and quadratic associations between CpG DNAm and age. Through a series of tests (described in the Methods section of the manuscript), we found that predictor performance was optimized with the inclusion of a reduced number of CpGs in training (feature pre-selection). Ultimately, the subset of CpGs that maximized predictor performance was that consisting of the 10,000 CpGs most associated to age linearly, as well as the 300 CpGs most associated to age quadratically. The resulting model, trained using a LOCO approach and considering just these pre-selected features, performed with r = 0.96, RMSE = 3.96, and MAE = 2.33 (**Additional file 2: Figure 3**).

**a)**

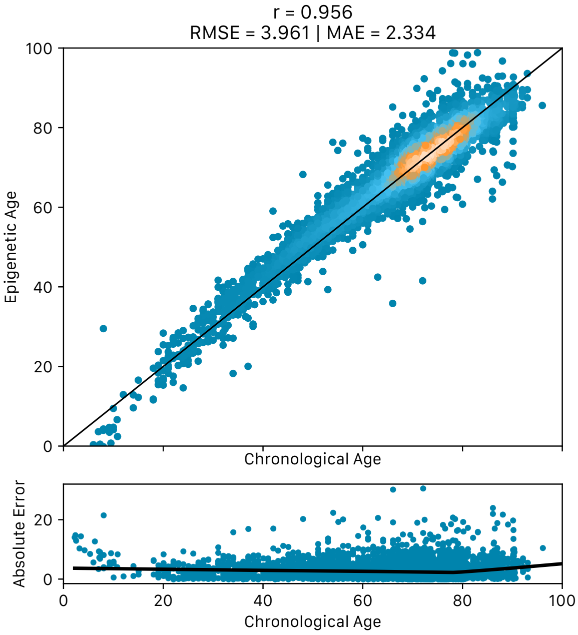


**b)**

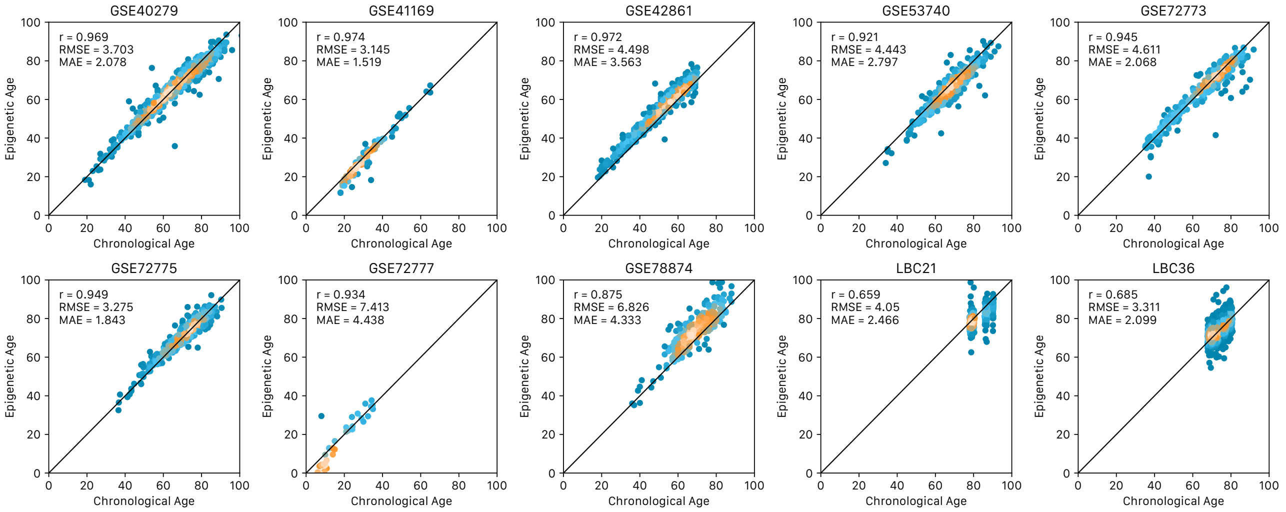


**Additional file 2: Figure 3. cAge predictor (trained using LOCO framework, on linear age, and with feature pre-selection) performance on 10 external testing datasets**, (a) across all datasets considered, and (b) per cohort. Performance metrics shown include Pearson correlation (r), root mean squared error (RMSE), and median absolute error (MAE).

## 4. LOCO with log(age), no feature pre-selection

To account for possible non-linear associations, we further explored training on both log(age) and linear age. A model trained on linear age was used as a starting point, and if a prediction of under 20 years old was returned, a second and final prediction was calculated using the model trained on log(age). As such, a model trained on all CpGs, considering a LOCO approach, and trained on both log(age) and linear age, performed with r = 0.93, RMSE = 5.25, and MAE = 3.43 (**Additional file 2: Figure 4**).

**a)**

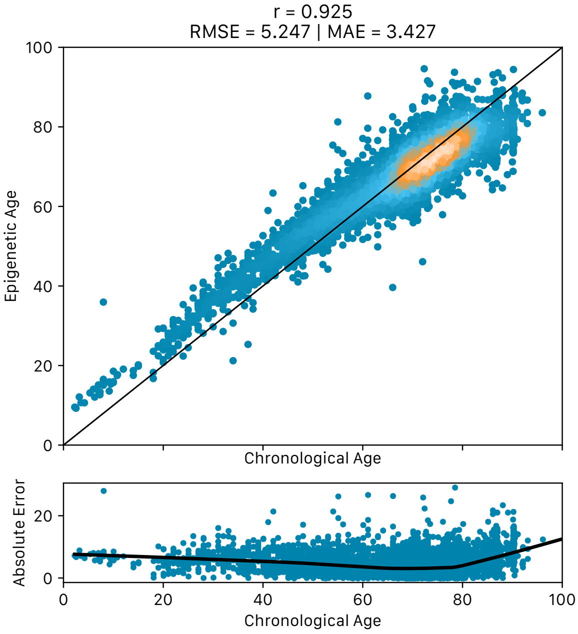


**b)**

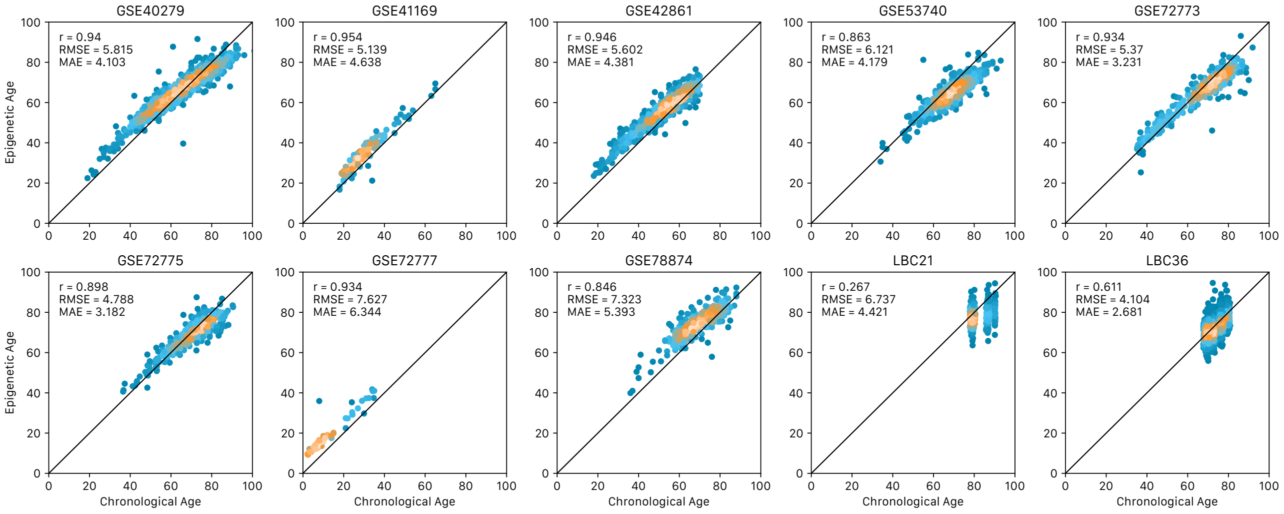


**Additional file 2: Figure 4. cAge predictor (trained using LOCO framework, on linear and log-transformed age for under 20s, and with no feature pre-selection) performance on 10 external testing datasets**, (a) across all datasets considered, and (b) per cohort. Performance metrics shown include Pearson correlation (r), root mean squared error (RMSE), and median absolute error (MAE).

## 5. LOCO with log(age), feature pre-selection

Having explored these different frameworks, we finally trained a model using a LOCO approach, training on both log(age) and linear age, and performing feature pre-selection ahead of elastic net. Ultimately, this model performed the best, and is presented as the final cAge model in our manuscript, performance showcased in **Figure 3**.

## bAge predictors

CpG-based predictors of mortality, in addition to the EpiScore-based predictor showcased in the main manuscript, were explored. Using the mortality EWAS results presented in the main manuscript, we generated subsets of CpGs which were used as training features for a direct CpG-to-mortality predictor, via elastic net Cox penalised regression (parameters used for these models and further details in Methods section of manuscript). Further, we explored the training of a predictor considering both subsets of CpGs as well as EpiScores as training features. GS data was used in training, whilst LBC1921 and LBC1936 data was used for testing.

Here, we describe the performance of these two predictors. Ultimately, we found that an EpiScore-based predictor associated more strongly with all-cause mortality than those explored here, hence our inclusion of the former in our manuscript.

## 1. EWAS based CpG predictor (EWASAge)

CpG subsets based on our mortality EWAS were considered (from 100 to 10,000 CpGs most associated - by P-value - with all-cause mortality). The resulting predictors for each of these subsets, regressed on age, were evaluated for their association with all-cause mortality using Cox models, adjusting for age and sex. We found that feature pre-selection to the top 800 most associated CpGs to mortality returned the best performing predictor considering all LBC samples, with Hazard Ratio (HR) = 1.38 [1.28, 1.48], p = 5.32 x 10-18. Prediction by cohort returned HRs of 1.36 [1.22, 1.51], p = 8.86 x 10-9 in LBC1921 and 1.40 [1.29, 1.59], p = 9.02 x 10-11 in LBC1936 (**Additional file 2: Figure 5**).

None of the CpG-based models outperformed the EpiScore-trained models, the 95% CI of which is shown in **Additional file 2: Figure 5** for LBC1921 (shaded in blue), and LBC1936 (shaded in orange).

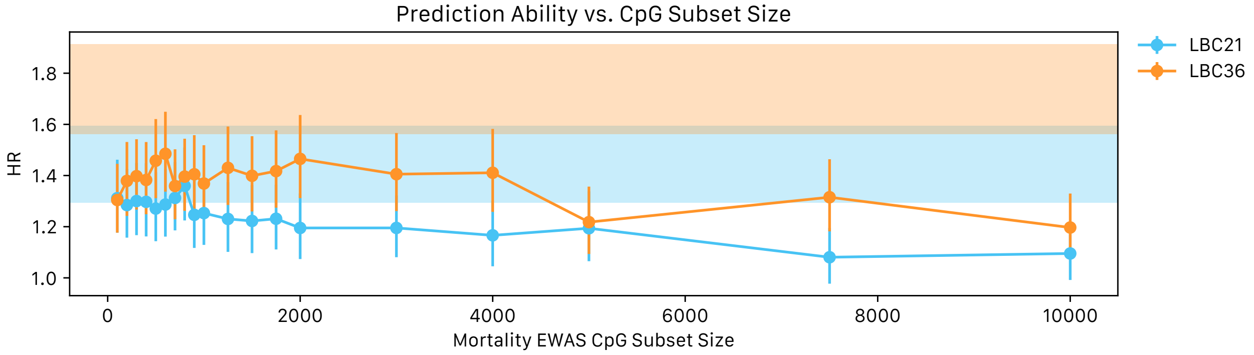


**Additional file 2: Figure 5. CpG-based bAge predictor performance, trained on different CpG subsets originating from mortality EWAS for each LBC cohort.** Dots correspond to HR estimates, with vertical lines indicating 95% CI. Shaded in blue and orange are the 95% CI of the HR in the EpiScore-based model for LBC1921 and LBC1936, respectively.

## 2. EWAS based CpG predictor + EpiScores (EWASAgeCombo)

In addition to a CpG-only based predictor of mortality, we explored a combined CpG-EpiScore approach, by which different subsets of CpGs, also originating from the mortality EWAS, were introduced as training features in addition to the EpiScores used in our final bAge predictor, described in the main manuscript. Prediction performance was assessed as before.

We found that the EpiScore-only model showed a stronger association with mortality in both LBC1921 and LBC1936. This is seen in **Additional file 2: Figure 6**, with the highest HR and 95% CI at the 0 CpG subset size mark.

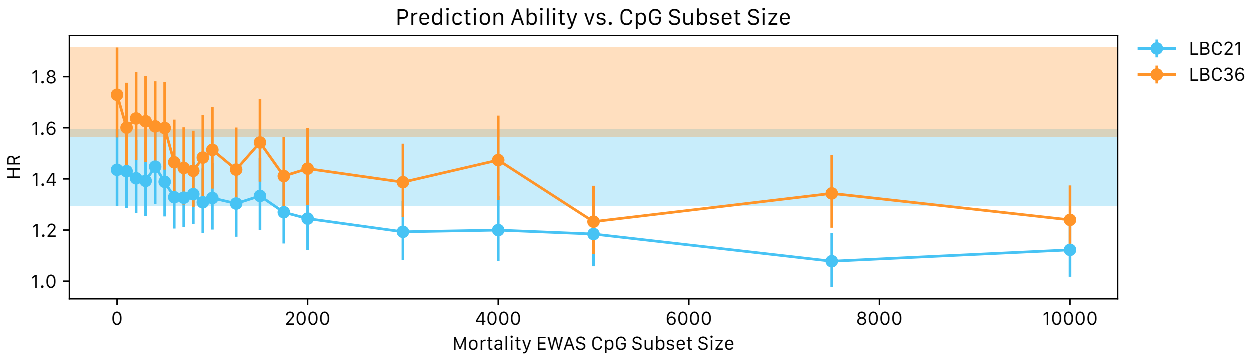


**Additional file 2: Figure 6. CpG-based bAge predictor performance, trained on different CpG subsets originating from mortality EWAS in addition to protein EpiScores, (a) considering all samples in LBC, (b) for each LBC cohort separately.** Dots correspond to HR estimates, with vertical lines indicating 95% CI. Shaded in blue and orange are the 95% CI of the HR in the EpiScore-based model for LBC1921 and LBC1936, respectively.
