# Supplementary material for: Refining epigenetic prediction of chronological and biological age
Source: Genome Med. 2023 Feb 28;15:12. doi: 10.1186/s13073-023-01161-y (PMC9976489; doi:10.1186/s13073-023-01161-y)

Supplementary Figures

Figure S1. Manhattan plots of (a) linear and (b) quadratic age EWAS in Generation Scotland. Dashed line indicates epigenome-wide significant level of $p = 3.6 \times 10^{-8}$. Genomic position indicated in x-axis, with -log10(*p*-value) of association indicated in y-axis. *p*-values capped at 10^{-320}.

(a)

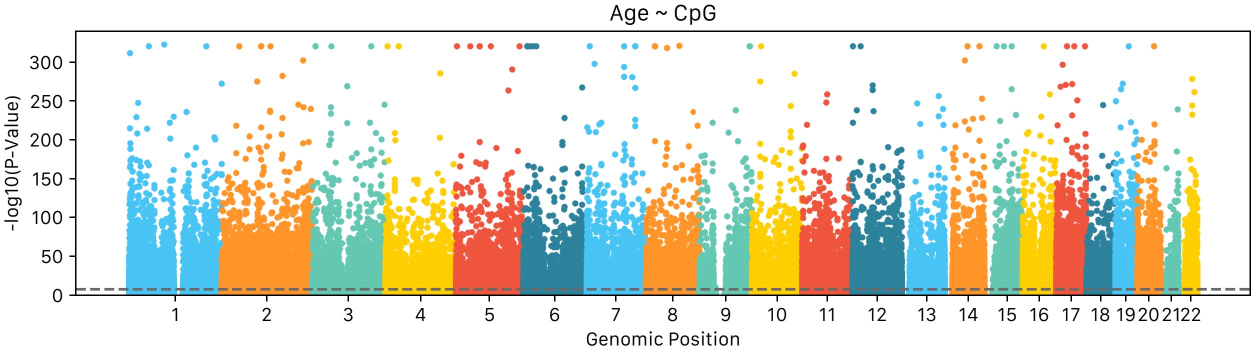

(b)

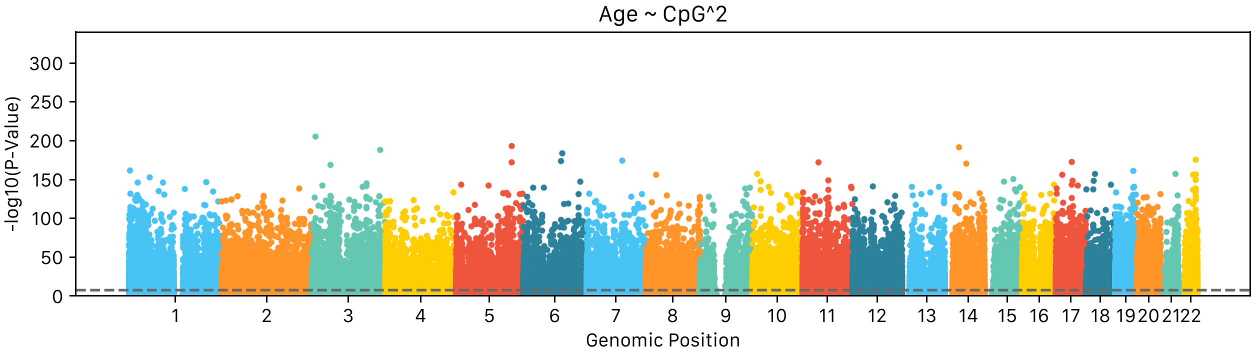

Figure S2. Scatterplots of top 15 associations from age ~ CpG EWAS. CpG beta values uncorrected for covariates (y-axis) are plotted against age (x-axis) for an unrelated subset of Generation Scotland participants (N = 4,450). LOESS curve with 95% confidence interval and contour plot overlaid. Gene names are taken from the Illumina EPIC array annotation file.

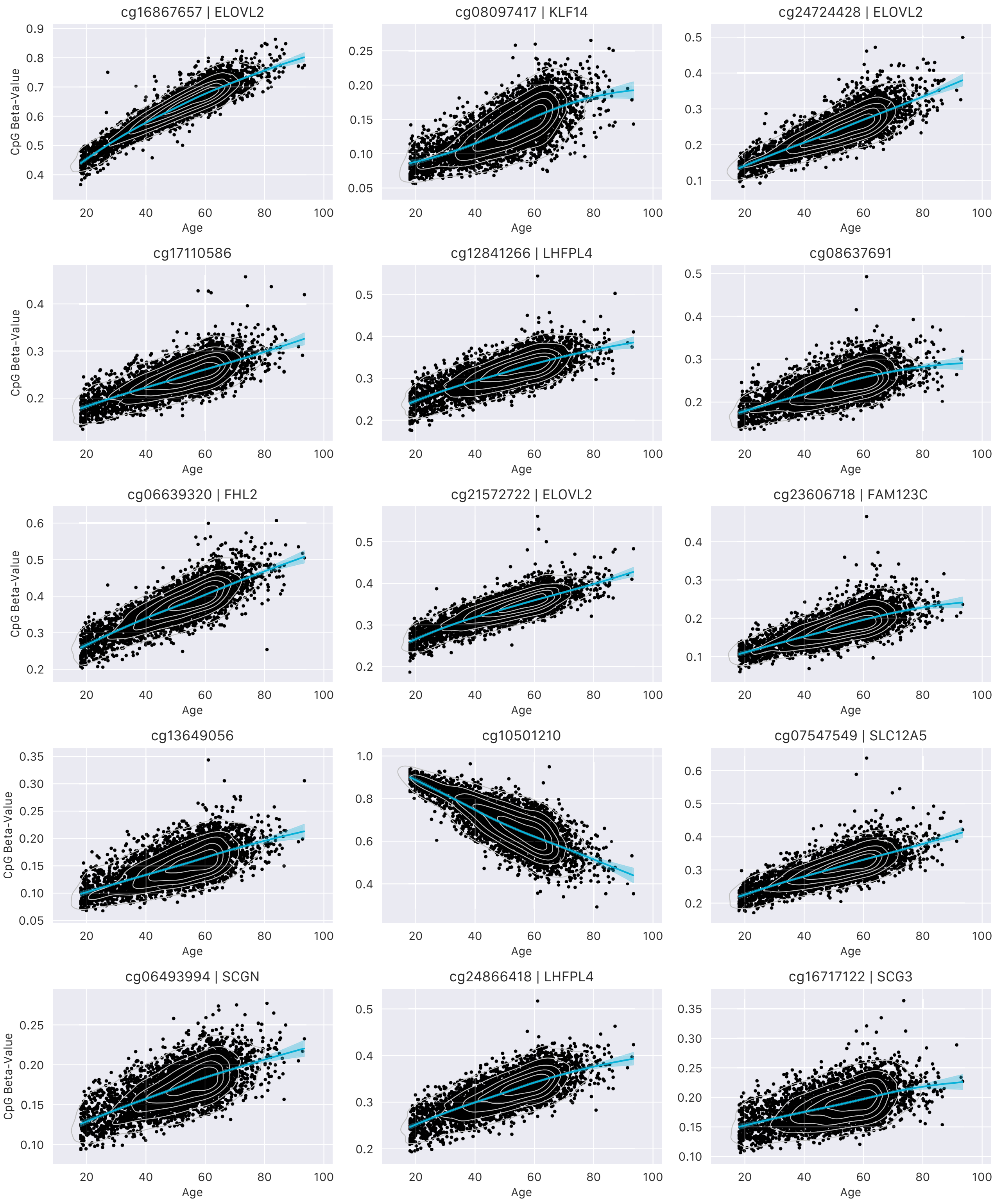

Figure S3. Scatterplots of top 15 associations from the age ~ CpG + CpG^2 EWAS. Significant associations are based on p-values obtained for the regression coefficient associated to CpG^2. CpG beta values uncorrected for covariates (y-axis) are plotted against age (x-axis) for an unrelated subset of Generation Scotland participants (N = 4,450). LOESS curve with 95% confidence interval and contour plot overlaid. Gene names are taken from the Illumina EPIC array annotation file.

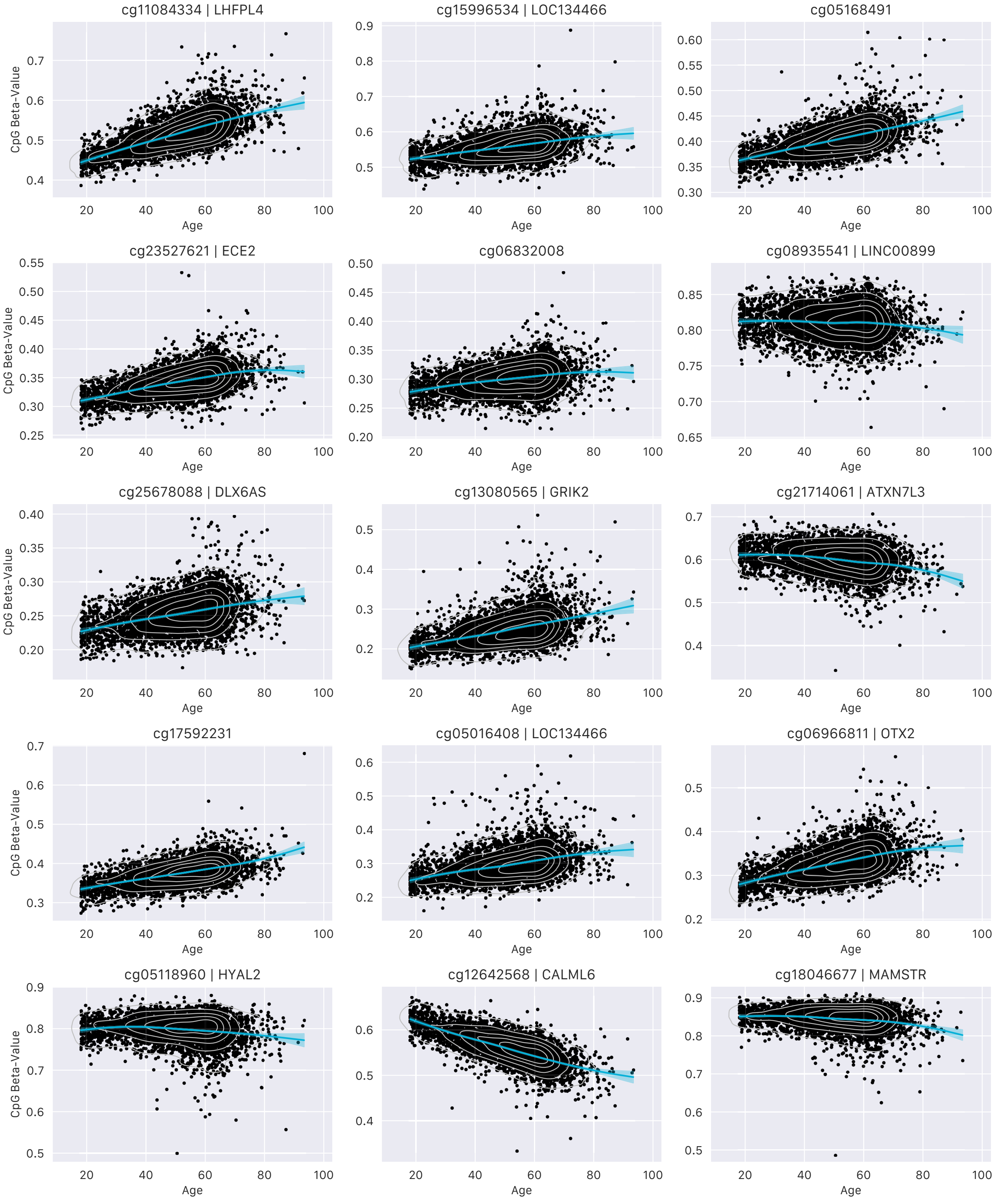

Figure S4. Manhattan plot of all-cause mortality EWAS in Generation Scotland. Dashed line indicates epigenome-wide significant level of $p = 3.6 \times 10^{-8}$. Genomic position indicated in x-axis, with -log10(*p*-value) of association indicated in y-axis.

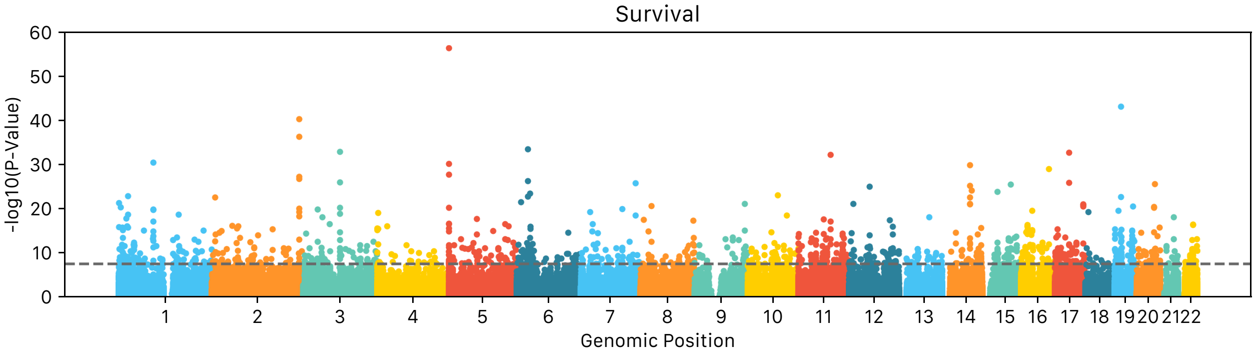

Figure S5. Comparison of Z-values for 200 overlapping epigenome-wide significant CpG-mortality associations reported by Colicino et al[4], and those considered in the present study (Pearson correlation, r). The grey line shows y = x (perfect correspondence). The correlations for the subset of findings from Colicino et al. with Z > 0 and Z < 0 are 0.29 and 0.46, respectively.

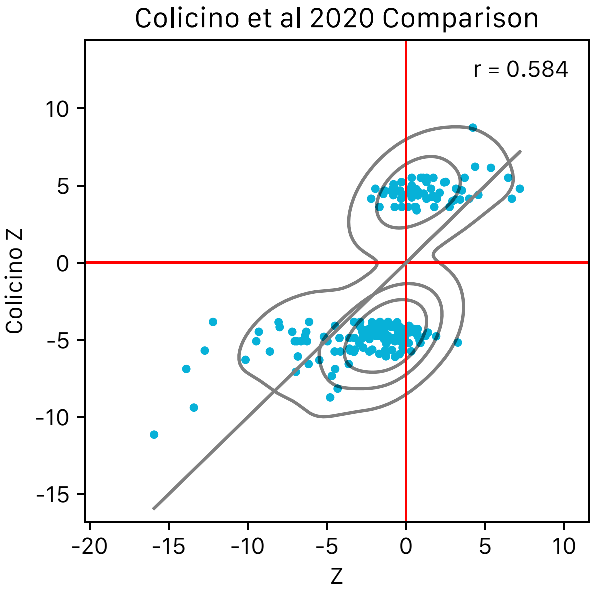

Figure S6. Prediction metrics (Pearson correlation - r, root mean square error - RMSE, and median absolute error - MAE) for GSE40279, as a function of CpGs included in training of a cAge predictor, trained using Generation Scotland data. CpG subsets obtained from linear age EWAS in Generation Scotland.

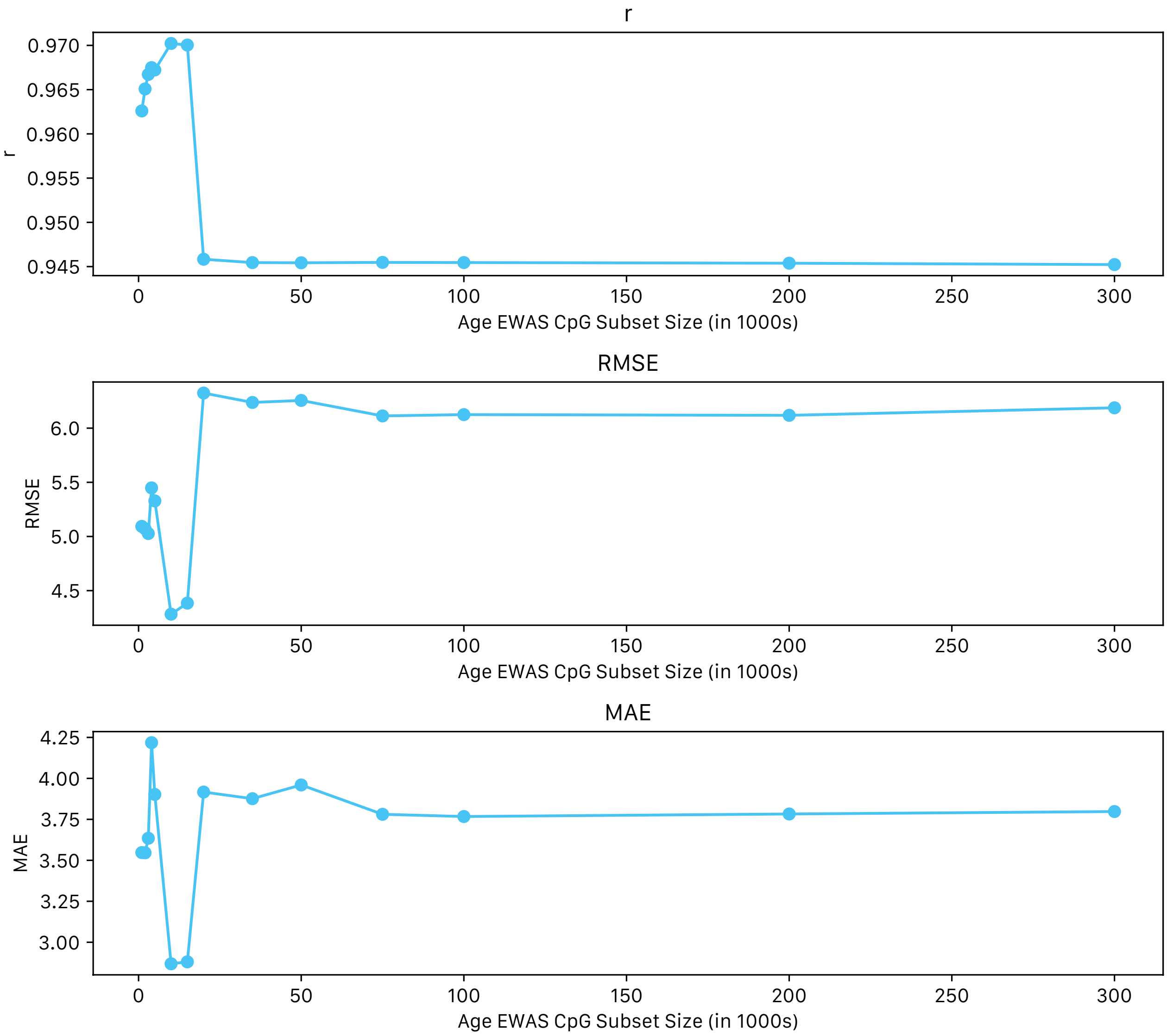

Figure S7. Prediction metrics (Pearson correlation - r, root mean square error - RMSE, and median absolute error - MAE) for GSE40279, as a function of CpG^2s included in training of a cAge predictor trained using Generation Scotland data, in addition to top 10K age-associated CpGs. CpG^2 subsets obtained from quadratic age EWAS in Generation Scotland.

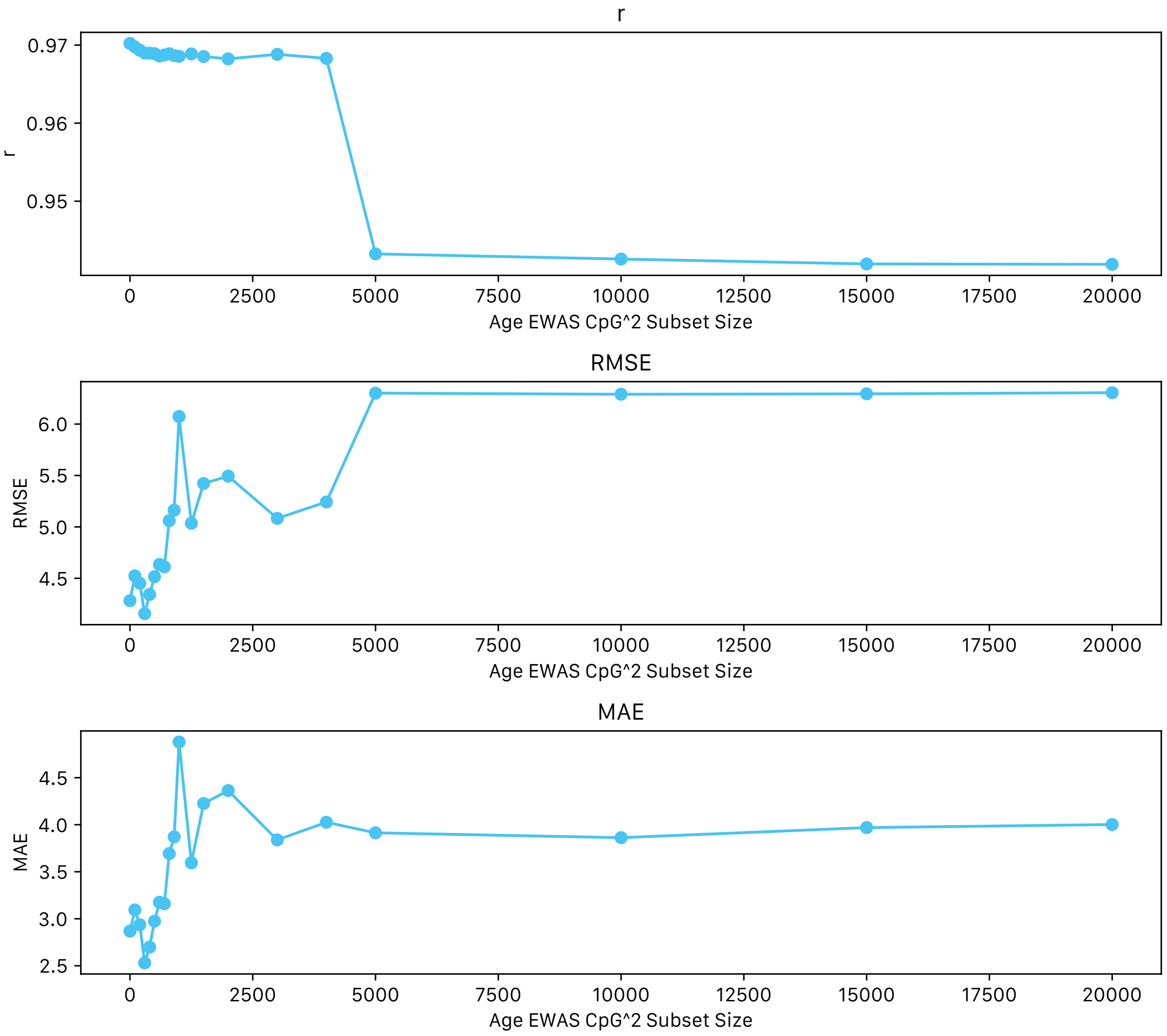

Figure S8. cAge predictor median absolute error (MAE) +/- 1.96 its standard deviation on 10 external testing datasets, as a function of age, in 10 year intervals.

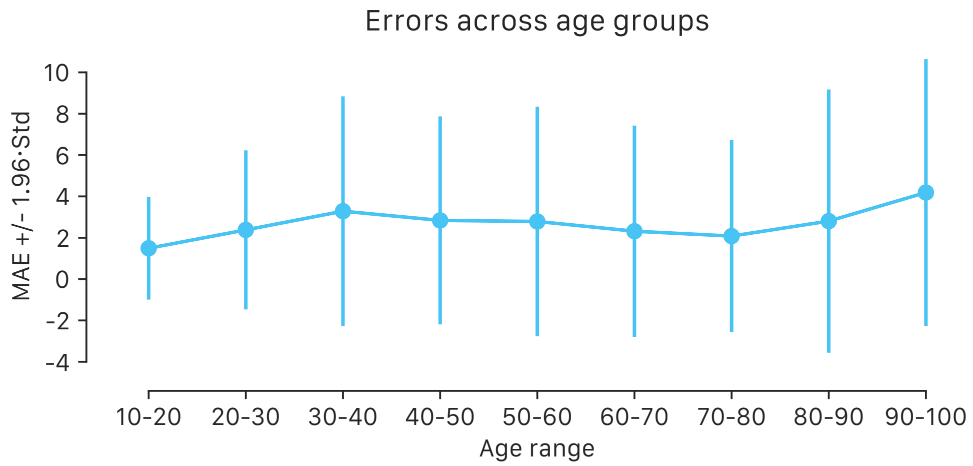

Figure S9. Forest plots of bAge, GrimAge, PhenoAge, and DunedinPACE predictors, applied to all-cause mortality in LBC1921 and LBC1936. Predictors regressed on age to obtain age acceleration residuals (AgeAccel). Hazard ratios are presented per standard deviation of the predictor variables, along with 95% confidence intervals. Cox models are adjusted for age at DNAm sampling and sex.

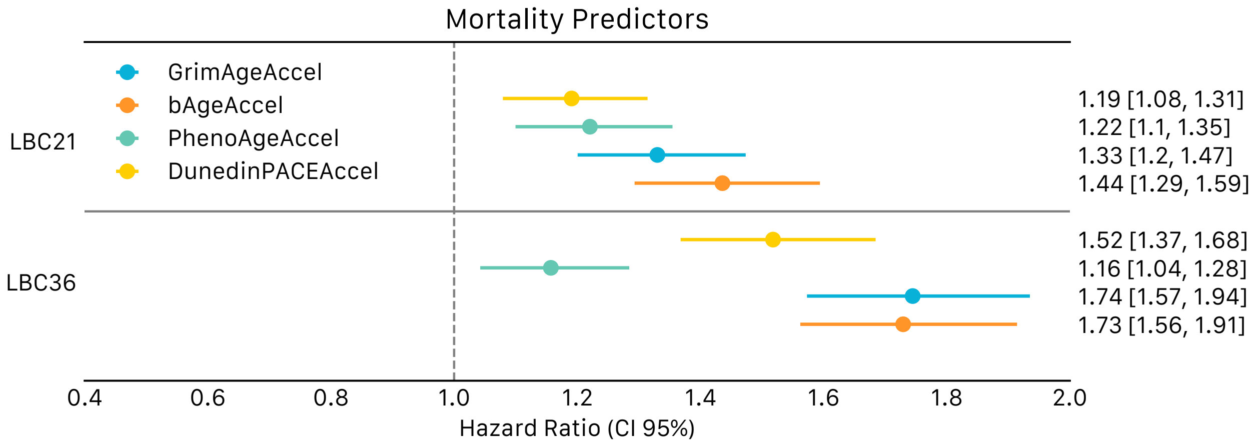

Supplement: Supplementary file 3 — Additional file 3. Figure S1. Manhattan plots of linear and quadratic age EWAS in Generation Scotland. Figure S2. Scatterplots of top 15 associations from age ~ CpG EWAS. Figure S3. Scatterplots of top 15 associations from the age ~ CpG + CpG2 EWAS. Figure S4. Manhattan plot of all-cause mortality EWAS. Figure S5. Comparison of Z-values for 200 overlapping epigenome-wide significant CpG-mortality associations reported by Colicino et al., and those considered in the present study. Figure S6. Prediction metrics for GSE40279, as a function of CpGs included in training of a cAge predictor, trained using Generation Scotland data. Figure S7. Prediction metrics for GSE40279, as a function of CpG2s included in training of a cAge predictor trained using Generation Scotland data, in addition to top 10K age-associated CpGs. Figure S8. cAge predictor median absolute error (MAE) +/- 1.96 its standard deviation on 10 external testing datasets, as a function of age, in 10 year intervals. Figure S9. Forest plots of bAge, GrimAge, PhenoAge, and DunedinPACE predictors, applied to all-cause mortality in LBC1921 and LBC1936. (PDF 12.2Mb). [file 13073_2023_1161_MOESM3_ESM.pdf]
